# Short‐ and Mid‐Term Outcomes of Proximal Gastrectomy With Double‐Tract Reconstruction Versus Total Gastrectomy in Early‐Stage Proximal Gastric Cancer

**DOI:** 10.1002/cam4.71258

**Published:** 2025-09-17

**Authors:** Ying Liu, Mingfang Yan, Zhaoyan Lin, Shenghong Wei, Yangming Li, Zhenmeng Lin, Xingfa Chen

**Affiliations:** ^1^ Department of Gastric Surgery Clinical Oncology School of Fujian Medical University, Fujian Cancer Hospital Fuzhou Fujian China; ^2^ Department of Anesthesiology Clinical Oncology School of Fujian Medical University, Fujian Cancer Hospital Fuzhou Fujian China; ^3^ College of Animal Science Fujian Agriculture and Forestry University Fuzhou Fujian China; ^4^ Department of Radiology Clinical Oncology School of Fujian Medical University, Fujian Cancer Hospital Fuzhou Fujian China

**Keywords:** double‐tract reconstruction, nutritional status, proximal gastric cancer, quality of life, total gastrectomy

## Abstract

**Background:**

Total gastrectomy (TG) is the predominant approach for proximal gastric cancer; however, it frequently leads to various postoperative nutritional and metabolic disorders. Recently, double‐tract reconstruction (DTR) following proximal gastrectomy (PG) has been developed to preserve gastric function and reduce postoperative reflux esophagitis. This study aimed to compare the outcomes of PG‐DTR with those of TG in patients with proximal gastric cancer.

**Methods:**

Patients with clinically staged cT1 to cT2 proximal gastric cancer who underwent either PG‐DTR or TG with Roux‐en‐Y reconstruction (TG‐RY) were enrolled. Propensity score matching (PSM) was applied to reduce confounding bias. Surgical outcomes, nutritional status, reflux esophagitis, and prognosis were prospectively analyzed. Quality of life (QOL) was assessed at 3, 6, and 12 months postoperatively using the Postgastrectomy Syndrome Assessment Scale‐45 (PGSAS‐45).

**Results:**

After PSM, 93 patients were included in each group (PG‐DTR and TG‐RY). The PG‐DTR group had fewer retrieved lymph nodes than the TG‐RY group. No significant differences were observed between the groups in operative time, blood loss, postoperative complications, length of hospital stay, reflux esophagitis, overall survival (OS), or disease‐free survival (DFS). Female sex, preoperative BMI, TG‐RY procedure, and severe postoperative complications (POCs) were identified as independent risk factors for malnutrition at 3 months postgastrectomy. Postoperatively, the TG‐RY group exhibited significantly lower levels of hemoglobin, total protein, and albumin than the PG‐DTR group. No differences were observed in postoperative QOL between the two groups, except for greater weight loss in the TG‐RY group.

**Conclusions:**

Compared to TG‐RY, PG‐DTR was associated with improved postoperative nutritional status, including better body weight, hemoglobin, and albumin levels, in patients with early‐stage proximal gastric cancer. Additionally, PG‐DTR demonstrated comparable short‐term outcomes, prognosis, and postoperative QOL to TG‐RY, further supporting its viability as a surgical option.

## 1 Introduction

Gastric cancer was the fifth most commonly diagnosed cancer and the fourth leading cause of cancer‐related mortality worldwide in 2020 [1]. In recent years, the incidence of tumors in the upper stomach, particularly adenocarcinomas of the esophagogastric junction (EGJ; predominantly Siewert type II and III tumors), has been steadily rising, likely due to factors such as the increasing prevalence of gastroesophageal reflux disease, changes in *Helicobacter pylori* infection rates, and environmental influences, including the rising rates of obesity [2, 3]. The incidence of early gastric cancer has also increased, primarily due to advancements in diagnostic technologies, improved screening methods, and greater public awareness [4, 5].

Total gastrectomy (TG) is the conventional surgical option for proximal gastric cancer, which enables comprehensive lymph node dissection and helps prevent severe reflux esophagitis. However, TG is linked to significant long‐term nutritional and metabolic complications, as well as reduced quality of life (QOL) [6, 7], which pose particular challenges for early gastric cancer patients with favorable prognoses and longer survival.

According to the 2018 Japanese gastric cancer treatment guidelines, proximal gastrectomy (PG) is recommended for early upper gastric cancer if an R0 resection can preserve more than half of the distal stomach [8]. PG aims to preserve partial gastric function but disrupts the anti‐reflux mechanism of the cardia while retaining the pylorus, which may delay gastric emptying. Consequently, PG is associated with complications such as severe reflux esophagitis and anastomotic strictures [9]. To mitigate these issues, various reconstruction techniques—including esophagogastrostomy, jejunal pouch interposition, jejunal interposition, and double‐tract reconstruction (DTR)—have been developed. Each reconstruction method has unique advantages and limitations; however, high‐quality evidence remains scarce, and no consensus on the optimal reconstruction technique has been established [10]. PG‐DTR provides a dual route for food passage through the residual stomach and jejunum, demonstrating effective anti‐reflux properties and gaining clinical popularity [11, 12]. While PG‐DTR has shown short‐term feasibility and safety, data on its mid‐term nutritional outcomes and QOL remain inconsistent [13, 14, 15, 16]. Thus, we conducted a comparative analysis of PG‐DTR and TG in patients with early‐stage proximal gastric cancer to provide further insight into their respective outcomes.

## 2 Method

### 2.1 Patients

**FIGURE 1:**
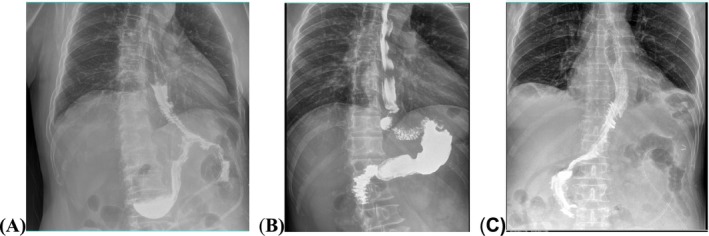
Postoperative fluoroscopy on day 5 following PG‐DTR. (A) Contrast agent flowing through both the remnant stomach and jejunal pathway into the distal jejunum. (B) Contrast agent flowing solely through the remnant stomach. (C) Contrast agent flowing solely through the jejunal pathway.

Patients with proximal gastric cancer were prospectively enrolled in this observational study from February 2021 to March 2023. The inclusion criteria were: (1) Histologically confirmed adenocarcinoma of the stomach; (2) Underwent PG‐DTR or TG with Roux‐en‐Y reconstruction (TG‐RY); (3) Tumor located in the upper third of the stomach, with a clinical stage of cT1 to cT2; (4) No severe comorbidities. The exclusion criteria were: (1) Siewert type I adenocarcinoma of the EGJ; (2) Presence of distant metastasis; (3) Tumor size > 4 cm; (4) Postoperative fluoroscopy showing contrast passage solely through the remnant stomach or only through the jejunum in the PG‐DTR group (as shown in Figure 1); (5) Incomplete clinicopathological data.

### 2.2 Definitions and Clinical Outcomes

Fluoroscopy is routinely performed on postoperative Day 5 for PG‐DTR patients. Follow‐up was conducted every 3 months in the first postoperative year and every 6 months in the subsequent second and third years, with the last follow‐up in April 2024. Disease‐free survival (DFS) was defined as the interval from surgery to the first occurrence of disease recurrence, last follow‐up, or death from any cause. Overall survival (OS) refers to the time from surgery to either death from any cause or the last follow‐up.

Routine endoscopy was performed approximately 1 year postoperatively to assess the severity of reflux esophagitis using the Los Angeles Classification System [17]. Pathological staging was conducted following the AJCC 8th edition staging system for gastric cancer [18].

Surgical outcomes assessed included blood loss, operative time, resected lymph nodes, radical resection status, postoperative complications (POCs), time to first flatus, time to first soft diet, and postoperative stay. POCs were classified according to the Clavien–Dindo grading system, with grades III to V defined as severe POCs [19]. Nutritional indicators measured were hemoglobin, total protein, albumin, and body mass index (BMI). Bowel function recovery was evaluated by the time to first flatus and the initiation of a soft diet [20].

QOL was assessed using the Postgastrectomy Syndrome Assessment Scale‐45 (PGSAS‐45), a multidimensional tool comprising 45 items. It includes 23 items grouped into seven symptom subscales: esophageal reflux, abdominal pain, meal‐related distress, indigestion, diarrhea, constipation, and dumping. Additionally, 22 items evaluate aspects such as decrease in body weight, ingested amount of food per meal, necessity for additional meals, quality of ingestion, ability for work, dissatisfaction with symptoms, meals, working, daily life, and both physical and mental component summaries. Overall, this comprehensive scale provides a thorough assessment of QOL after gastrectomy [21, 22, 23].

The GLIM (Global Leadership Initiative on Malnutrition) criteria were applied to assess malnutrition at the third postoperative month. This evaluation followed a two‐step process: first, the Nutrition Risk Screening (NRS‐2002) was used to identify at‐risk patients, followed by the GLIM criteria to confirm malnutrition, requiring at least one phenotypic criterion (e.g., weight loss, low BMI, or reduced muscle mass) and one etiologic criterion (e.g., reduced food intake, inflammation, or disease burden). Since all patients underwent gastrectomy for gastric cancer, they inherently met the etiologic criterion due to disease burden and inflammation [24, 25].

### 2.3 Detailed Surgical Technique of PG‐DTR

**FIGURE 2:**
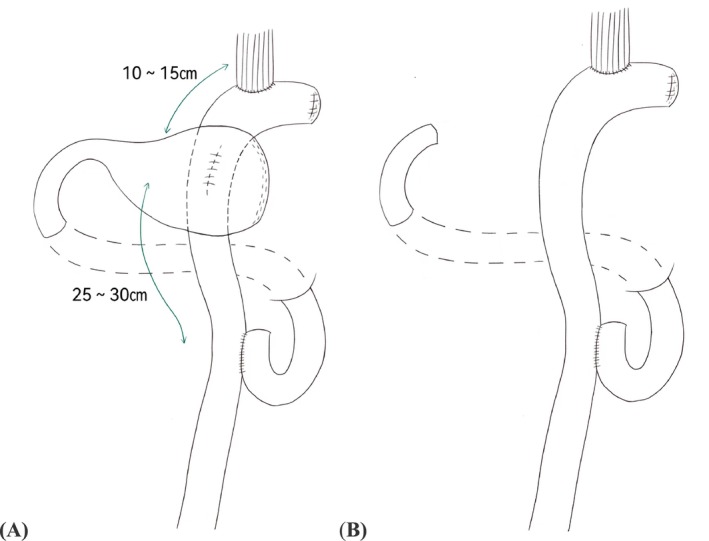
Schematic of reconstruction. (A) Proximal gastrectomy with double‐tract reconstruction. (B) Total gastrectomy with Roux‐en‐Y reconstruction.

In accordance with the Japanese Gastric Cancer Treatment Guidelines, a D1+ lymphadenectomy was performed, targeting lymph nodes No. 1, 2, 3a, 4sa, 4sb, 7, 8a, 9, and 11p [26]. The procedure included transection of the esophagus and resection of the tumor with the proximal stomach. The jejunum was divided approximately 20 to 25 cm distal to the ligament of Treitz. An end‐to‐side esophagojejunostomy was performed using a circular stapler, and the jejunal stump was closed with a linear stapler. A side‐to‐side jejunojejunostomy was created approximately 45 to 50 cm distal to the esophagojejunostomy between the proximal and distal jejunum. Additionally, a side‐to‐side anastomosis was performed between the jejunum and the posterior wall of the remnant stomach using a 60‐mm linear stapler, 10 to 15 cm distal to the esophagojejunostomy, followed by closure of the gastric stump (Figure 2).

### 2.4 Statistical Analysis

All statistical analyses were performed using SPSS version 27.0 and R version 4.1.1. Categorical variables were analyzed using the chi‐squared test or Fisher's exact test, while continuous variables were compared with either Student's *t*‐test or the Mann–Whitney *U* test, depending on data distribution. To reduce confounding, propensity score matching (PSM) was performed using one‐to‐one nearest neighbor matching without replacement with a caliper width of 0.02. The propensity score was estimated using a logistic regression model that included the following covariates: age, sex, preoperative BMI, hypertension, diabetes, preoperative hemoglobin, preoperative serum iron, preoperative total protein, preoperative albumin, ASA score, tumor size, type of surgery, pTNM stage, and adjuvant chemotherapy. Multivariable logistic regression analysis was then conducted to identify independent risk factors for malnutrition. Longitudinal changes between groups were analyzed using a linear mixed model (LMM), with Bonferroni correction applied to adjust for multiple comparisons. Survival analysis was conducted with the Kaplan–Meier method, and survival rates were compared using the Log‐rank test. A *p*‐value of < 0.05 was considered statistically significant.

## 3 Results

### 3.1 Patient Characteristics

**FIGURE 3:**
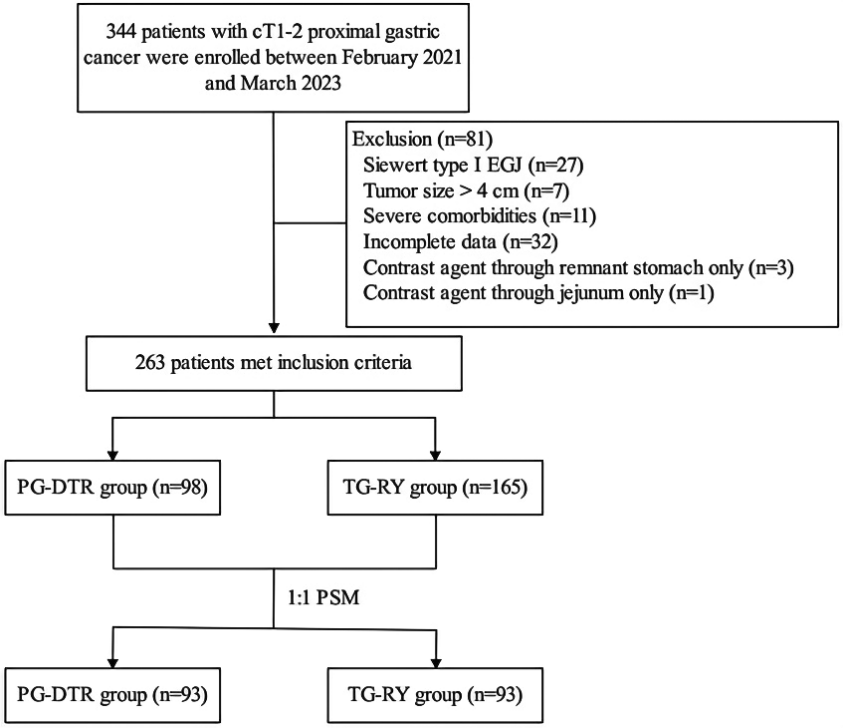
Flow chart of patient selection.

A total of 344 patients with clinical stage cT1 to cT2 proximal gastric cancer were initially enrolled. After applying the exclusion criteria, 263 patients were eligible for analysis, comprising 98 patients in the PG‐DTR group and 165 in the TG‐RY group (Figure 3). Before PSM, significant differences were observed in several baseline characteristics, including preoperative BMI, hypertension status, preoperative total protein and albumin levels, and tumor differentiation (*p* < 0.05). After 1:1 PSM, 93 matched patients were included in each group, resulting in a balanced cohort with no statistically significant differences in baseline characteristics, thus facilitating robust comparative analysis (*p* > 0.05; Table 1). After matching, all 186 patients completed postoperative assessments for body mass index, blood tests, and endoscopic examinations as scheduled. QOL at 3 months post‐surgery was evaluated using the PGSAS‐45 for all patients. However, three patients in the PG‐DTR group and five patients in the TG‐RY group missed the QOL assessment at 6 months post‐surgery. At the 12‐month follow‐up, seven patients in the PG‐DTR group and eight in the TG‐RY group did not complete the QOL assessment.

**TABLE 1:** Baseline characteristics of PG‐DTR and TG‐RY Groups before and after PSM.

	Before PSM			After PSM		
	PG‐DTR (*n* = 98)	TG‐RY (*n* = 165)	*p*	PG‐DTR (*n* = 93)	TG‐RY (*n* = 93)	*p*
Age (years), median (P_25_, P_75_)	60 (51, 66)	59 (50, 68)	0.809	60 (51, 67)	58 (50, 66)	0.440
Sex, *N* (%)						
Male	65 (66.3)	118 (71.5)	0.377	63 (67.7)	65 (69.9)	0.752
Female	33 (33.7)	47 (28.5)		30 (32.3)	28 (30.1)	
Preoperative BMI (kg/m^2^), mean ± SD	23.3 ± 2.6	22.3 ± 2.6	0.018	23.2 ± 2.6	23.3 ± 3.3	0.838
Previous abdominal operation, *N* (%)						
Yes	13 (13.3)	17 (10.3)	0.465	13 (14.0)	11 (11.8)	0.662
No	85 (86.7)	148 (89.7)		80 (86.0)	82 (88.2)	
Smoking history, *N* (%)						
Yes	25 (25.5)	33 (20.0)	0.297	23 (24.7)	24 (25.8)	0.866
No	73 (74.5)	132 (80.0)		70 (75.3)	69 (74.2)	
Alcohol history, *N* (%)						
Yes	31 (31.6)	41 (24.8)	0.233	28 (30.1)	31 (33.3)	0.636
No	67 (68.4)	124 (75.2)		65 (69.9)	62 (66.7)	
Hypertension, *N* (%)						
Yes	22 (22.4)	57 (34.5)	**0.039**	22 (23.7)	29 (31.2)	0.250
No	76 (77.6)	108 (65.5)		71 (76.3)	64 (68.8)	
Diabetes, *N* (%)						
Yes	19 (19.4)	22 (13.3)	0.191	16 (17.2)	19 (20.4)	0.574
No	79 (80.6)	143 (86.7)		77 (82.8)	74 (79.6)	
Preoperative hemoglobin (g/L), median (P_25_, P_75_)	129.5 (119.0, 138.3)	133.0 (120.0, 146.0)	0.154	129.0 (118.5, 138.0)	135.0 (123.0, 144.5)	0.121
Preoperative serum iron (μmol/L), median (P_25_, P_75_)	20.1 (13.6, 26.4)	21.7 (15.3, 27.4)	0.151	20.0 (13.6, 26.5)	21.0 (13.7, 26.8)	0.778
Preoperative total protein (g/L), median (P_25_, P_75_)	69.3 (64.2, 76.5)	67.2 (62.4, 73.8)	**0.031**	69.3 (64.2, 76.1)	69.5 (64.8, 75.1)	0.739
Preoperative albumin (g/L), median (P_25_, P_75_)	41.8 (38.2, 47.1)	39.5 (36.7, 44.7)	**0.022**	41.5 (38.0, 47.2)	41.2 (38.5, 47.0)	0.958
CEA (ng/mL), *N* (%)						
≤ 5	90 (91.8)	148 (89.7)	0.567	86 (92.5)	86 (92.5)	1.000
> 5	8 (8.2)	17 (10.3)		7 (7.5)	7 (7.5)	
CA19‐9 (U/mL), *N* (%)						
≤ 30	93 (94.9)	152 (92.1)	0.389	88 (94.6)	86 (92.5)	0.551
> 30	5 (5.1)	13 (7.9)		5 (5.4)	7 (7.5)	
ASA score, *N* (%)						
I	80 (81.6)	126 (76.4)	0.595	75 (80.6)	75 (80.6)	0.942
II	12 (12.2)	25 (15.2)		12 (12.9)	11 (11.8)	
III	6 (6.1)	14 (8.5)		6 (6.5)	7 (7.5)	
Tumor size (cm), mean ± SD	2.5 ± 1.1	2.6 ± 1.0	0.549	2.5 ± 1.1	2.5 ± 1.0	0.910
Differentiation, *N* (%)						
Well/moderate	38 (38.8)	131 (79.4)	**0.001**	35 (37.6)	37 (39.8)	0.763
Poor/undifferentiated	60 (61.2)	15 (9.1)		58 (62.4)	56 (60.2)	
Borrmann type, *N* (%)						
I	13 (13.3)	15 (9.1)	0.330	13 (14.0)	11 (11.8)	0.740
II	48 (49.0)	74 (44.8)		45 (48.4)	42 (45.2)	
III	37 (37.8)	76 (46.1)		35 (37.6)	40 (43.0)	
Siewert type, *N* (%)						
II	47 (48.0)	74 (44.8)	0.625	43 (46.2)	40 (43.0)	0.658
III	51 (52.0)	91 (55.2)		50 (53.8)	53 (57.0)	
Type of surgery, *N* (%)						
Open	8 (8.2)	17 (10.3)	0.769	8 (8.6)	9 (9.7)	0.819
Laparoscopic	83 (84.7)	134 (81.2)		78 (83.9)	79 (84.9)	
Robotic	7 (7.1)	14 (8.5)		7 (7.5)	5 (5.4)	
Reconstruction method, *N* (%)						
Intracorporeal	26 (26.5)	33 (20.0)	0.220	22 (23.7)	27 (29.0)	
Extracorporeal	72 (73.5)	132 (80.0)		71 (76.3)	66 (71.0)	
pTNM stage^a^, *N* (%)						
I	80 (81.6)	126 (76.4)	0.316	75 (80.6)	74 (79.6)	0.854
II	18 (18.4)	39 (23.6)		18 (19.4)	19 (20.4)	
Adjuvant chemotherapy, *N* (%)						
Yes	16 (16.3)	36 (21.8)	0.280	16 (17.2)	20 (21.5)	0.458
No	82 (87.7)	129 (78.2)		77 (82.8)	73 (78.5)	

*Note:* The bolded values in Table 1 indicate statistically significant differences (*p* < 0.05).

Abbreviations: BMI, body mass index; PG‐DTR, proximal gastrectomy with double‐tract reconstruction; PSM, propensity score matching; SD, standard deviation; TG‐RY, total gastrectomy with Roux‐en‐Y reconstruction.

^a^ TNM staging according to the UICC TNM staging 8th edition.

### 3.2 Surgical Outcomes

The TG‐RY group had a significantly greater total number of resected lymph nodes than the PG‐DTR group (*p* < 0.05). This difference was attributed to the additional retrieval of lymph nodes at stations #4d, 5, 6, and 12a in the TG‐RY procedure—stations that are not routinely dissected in PG‐DTR according to current guidelines. However, the number of lymph nodes retrieved from stations #1 to 3, 4sa, 4sb, 7 to 9, and 11 did not differ significantly between the two groups (*p* > 0.05). Importantly, none of the additionally retrieved lymph nodes (#4d, 5, 6, 12a) in the TG‐RY group were positive for metastasis. No other significant differences were observed in surgical outcomes between the two groups (*p* > 0.05; Table 2). Additionally, as shown in Table 3, there were no significant differences in overall postoperative complications (POCs), severe POCs, or length of hospital stay between the two groups across all subgroups (*p* > 0.05).

**TABLE 2:** Comparison of short‐term outcomes between PG‐DTR and TG‐RY Groups.

	PG‐DTR (*n* = 93)	TG‐RY (*n* = 93)	*p*
Operation time (min), mean ± SD	166.4 ± 33.8	173.8 ± 37.2	0.161
Estimated blood loss (mL), mean ± SD	204.4 ± 82.7	222.8 ± 124.1	0.236
Resected lymph nodes, mean ± SD			
#4d, 5, 6, 12a	—	9.6 ± 4.2	
#1–3, 4sa, 4sb, 7–9,11	27.3 ± 8.5	25.6 ± 17.0	0.372
Total	27.3 ± 8.5	35.1 ± 17.0	**0.004**
No. of positive lymph nodes, median (P_25_, P_75_)			
#4d, 5, 6, 12a	—	0	
#1–3, 4sa, 4sb, 7–9,11	0 (0, 1)	0 (0, 0)	0.120
Radical resection, *N* (%)			
R0	92 (98.9)	90 (96.8)	0.312
R1	1 (1.1)	3 (3.2)	
Intraoperative blood transfusion, *N* (%)			
Yes	10 (10.8)	4 (4.3)	0.095
No	83 (89.2)	89 (95.7)	
Time to first flatus (days), mean ± SD	4.2 ± 2.7	4.6 ± 3.5	0.339
Time to first soft diet (days), mean ± SD	6.4 ± 2.7	6.7 ± 3.5	0.478
Postoperative stay (days), mean ± SD	11.7 ± 4.9	12.6 ± 5.3	0.256
Overall POCs, *N* (%)			
Yes	22 (23.7)	29 (31.2)	0.250
No	71 (76.3)	64 (68.8)	
Severe POCs, *N* (%)			
Yes	6 (6.5)	10 (10.8)	0.296
No	87 (93.5)	83 (89.2)	
Anastomotic leakage, *N* (%)			
Yes	2 (2.2)	6 (6.5)	0.148
No	91 (97.8)	87 (93.5)	
In‐hospital mortality, *N* (%)			
Yes	0 (0.0)	1 (1.1)	0.316
No	93 (100.0)	92 (98.9)	

*Note:* The bolded values in Table 2 indicate statistically significant differences (*p* < 0.05).

Abbreviations: PG‐DTR, proximal gastrectomy with double‐tract reconstruction; POCs, postoperative complications; SD, standard deviation; TG‐RY, total gastrectomy with Roux‐en‐Y reconstruction.

**TABLE 3:** Subgroup analyses of short‐term outcomes between PG‐DTR and TG‐RY groups.

		Patients	Overall POCs		Severe POCs		Hospital stay (days)	
			No.	*p*	No.	*p*	POD	*p*
Age (years), *N* (%)								
< 60	PG‐DTR	45 (48.4)	6 (13.3)	0.218	3 (6.7)	0.410	12.3 ± 6.5	0.859
	TG‐RY	52 (55.9)	12 (23.1)		6 (11.5)		12.5 ± 4.8	
≥ 60	PG‐DTR	48 (51.6)	16 (33.3)	0.429	3 (6.3)	0.540	11.1 ± 2.5	0.122
	TG‐RY	41 (44.1)	17 (41.5)		4 (9.8)		12.6 ± 5.9	
Sex, *N* (%)								
Male	PG‐DTR	63 (67.7)	17 (27.0)	0.510	4 (6.3)	0.544	11.7 ± 5.4	0.268
	TG‐RY	65 (69.9)	21 (32.3)		6 (9.2)		12.8 ± 5.5	
Female	PG‐DTR	30 (32.3)	5 (16.7)	0.277	2 (6.7)	0.545	11.8 ± 3.6	0.761
	TG‐RY	28 (30.1)	8 (28.6)		4 (14.3)		12.1 ± 4.9	
Siewert type, *N* (%)								
II	PG‐DTR	39 (41.9)	5 (12.8)	0.103	1 (2.6)	0.122	13.3 ± 3.9	0.109
	TG‐RY	44 (47.3)	12 (27.3)		5 (11.4)		15.2 ± 6.3	
III	PG‐DTR	54 (58.1)	17 (31.5)	0.729	5 (9.3)	0.872	10.6 ± 5.2	0.653
	TG‐RY	49 (52.7)	17 (34.7)		5 (10.2)		10.2 ± 2.5	

Abbreviations: PG‐DTR, proximal gastrectomy with double‐tract reconstruction; POCs, postoperative complications; day; POD, postoperative; TG‐RY, total gastrectomy with Roux‐en‐Y reconstruction.

### 3.3 Mid‐Term Outcomes

At 12 months postoperatively, no significant difference in reflux esophagitis incidence was observed between the two groups (*p* > 0.05). At 3 months postoperatively, a greater proportion of patients in the TG‐RY group experienced moderate to severe malnutrition compared to the PG‐DTR group (*p* < 0.05; Table 4). Multivariate analysis revealed that female sex, low preoperative BMI, TG‐RY reconstruction, and severe POCs were independent risk factors for malnutrition (*p* < 0.05; Table 5).

**TABLE 4:** Comparison of endoscopic reflux esophagitis and malnutrition between PG‐DTR and TG‐RY groups.

	PG‐DTR (*n* = 93)	TG‐RY (*n* = 93)	*p*
Reflux esophagitis^a^, *N* (%)			
No	87 (93.5)	89 (95.7)	0.567
Grade A	5 (6.5)	4 (4.3)	
Grade B	1 (0.0)	0 (0)	
Malnutrition^b^, *N* (%)			
No	80 (86.0)	61 (65.6)	**0.001**
Moderate	10 (10.8)	25 (26.9)	
Severe	3 (3.2)	7 (7.5)	

*Note:* The bolded values in Table 4 indicate statistically significant differences (*p* < 0.05).

Abbreviations: PG‐DTR, proximal gastrectomy with double‐tract reconstruction; TG‐RY, total gastrectomy with Roux‐en‐Y reconstruction.

^a^ Twelve months postoperatively.

^b^ Three months postoperatively.

**TABLE 5:** Univariate and multivariate logistic regression of malnutrition at 3 months postgastrectomy.

	Univariable analysis		Multivariable analysis	
	Odds ratio (95% CI)	*p*	Odds ratio (95% CI)	*p*
Age (years)	1.048 (1.013,1.085)	**0.007**	1.011 (0.962,1.063)	0.669
Sex				
Male	Reference		Reference	
Female	2.790 (1.390, 5.598)	**0.004**	5.526 (2.173, 14.052)	**< 0.001**
Preoperative BMI	0.723 (0.624, 0.838)	**< 0.001**	0.763 (0.646, 0.901)	**0.001**
Previous abdominal operation				
Yes	Reference			
No	0.744 (0.287, 1.929)	0.543		
Smoking history				
Yes	Reference			
No	0.677 (0.322, 1.421)	0.302		
Alcohol history				
Yes	Reference			
No	0.699 (0.346, 1.411)	0.317		
Hypertension				
Yes	Reference			
No	1.697 (0.751, 3.833)	0.203		
Diabetes				
Yes	Reference			
No	0.536 (0.241, 1.190)	0.125		
Preoperative hemoglobin (g/L)	0.993 (0.976, 1.010)	0.409		
Preoperative serum iron (μmol/L)	1.003 (0.956, 1.052)	0.898		
Preoperative total protein (g/L)	0.983 (0.945, 1.022)	0.394		
Preoperative albumin (g/L)	0.954 (0.904, 1.007)	0.086		
CEA (ng/mL)				
≤ 5	Reference			
> 5	1.278 (0.381, 4.292)	0.691		
CA19‐9 (U/mL)				
≤ 30	Reference			
> 30	0.609 (0.128, 2.890)	0.533		
ASA score				
I	Reference			
II	1.551 (0.589, 4.086)	0.374		
III	2.216 (0.679, 7.228)	0.187		
Tumor size (cm)	1.306 (0.938, 1.817)	0.114		
Differentiation				
Well/moderate	Reference			
Poor/undifferentiated	0.824 (0.416, 1.631)	0.579		
Borrmann type				
I	Reference			
II	0.597 (0.223, 1.598)	0.305		
III	0.586 (0.214, 1.603)	0.298		
Siewert type				
II	Reference			
III	1.641 (0.820, 3.285)	0.162		
Surgical option				
PG‐DTR	Reference		Reference	
TG‐RY	3.228 (1.563, 6.670)	**0.002**	6.438 (1.950, 21.252)	**0.002**
Type of surgery				
Open	Reference			
Laparoscopic	1.074 (0.331, 3.488)	0.905		
Robotic	0.650 (0.098, 4.290)	0.655		
Reconstruction method				
Intracorporeal	Reference			
Extracorporeal	1.140 (0.525, 2.475)	0.740		
pTNM stage^a^				
I	Reference			
II	0.546 (0.212, 1.408)	0.210		
Adjuvant chemotherapy				
Yes	Reference			
No	1.773 (0.802, 3.919)	0.157		
Overall POCs				
Yes	Reference			
No	0.598 (0.291, 1.230)	0.162		
Severe POCs				
Yes	Reference		Reference	
No	0.209 (0.073, 0.600)	**0.004**	0.087 (0.019, 0.390)	**0.001**

*Note:* The bolded values in Table 5 indicate statistically significant differences (*p* < 0.05).

Abbreviations: BMI, body mass index; PG‐DTR, proximal gastrectomy with double‐tract reconstruction; POCs, postoperative complications; TG‐RY, total gastrectomy with Roux‐en‐Y reconstruction.

^a^ TNM staging according to the UICC TNM staging 8th edition.

**FIGURE 4:**
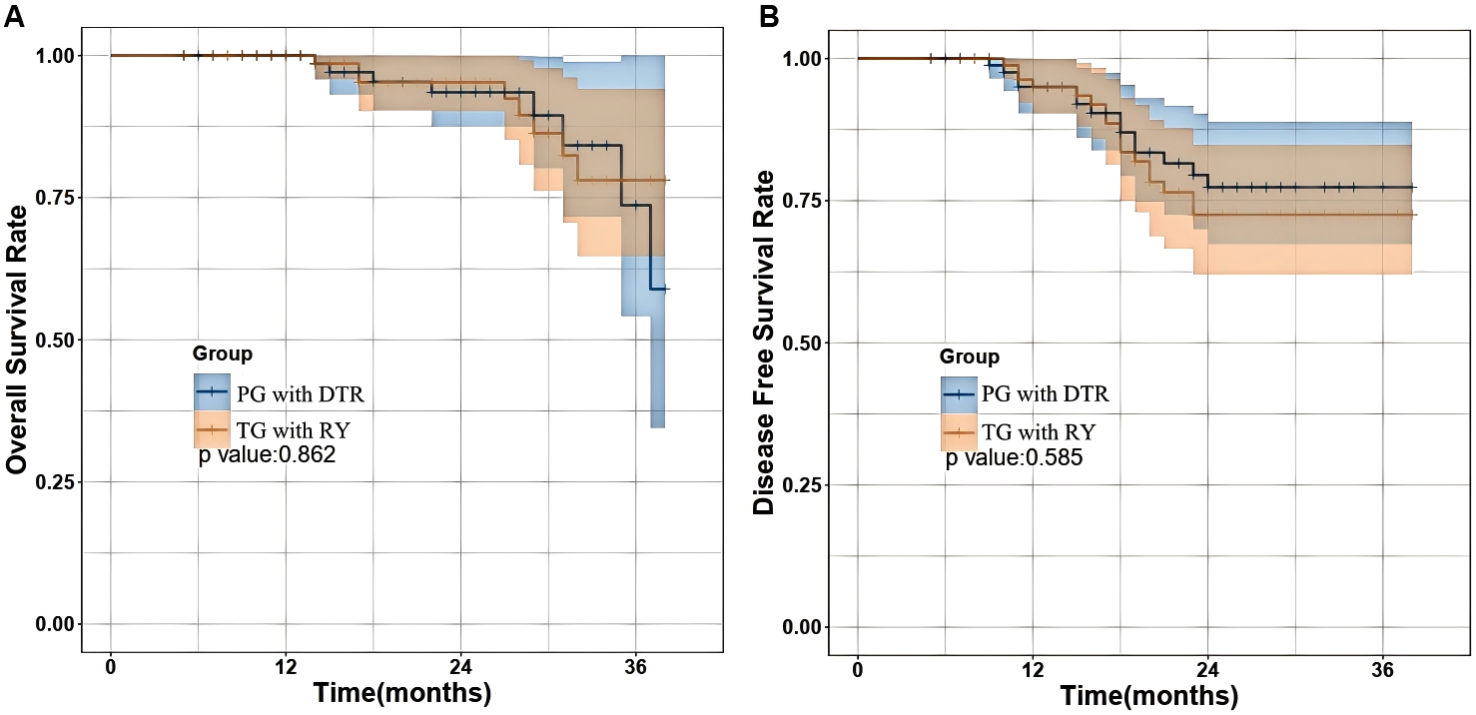
Kaplan–Meier curves comparing survival outcomes. (A) Overall survival; (B) Disease‐free survival.

No statistically significant differences in OS and DFS were observed between the PG‐DTR group and the TG‐RY group (*p* > 0.05; Figure 4).

Throughout this longitudinal study, hemoglobin, total protein, and serum albumin levels were higher in the PG‐DTR group compared to the TG‐RY group (*p* < 0.05). Specifically, hemoglobin levels in the PG‐DTR group were significantly higher at 3, 6, and 12 months postoperatively, while total protein and serum albumin levels were higher at 3 and 12 months postoperatively (*p* < 0.05; Figures S1 and S2).

Apart from the greater decrease in body weight observed in the TG‐RY group throughout the postoperative follow‐up period, the QOL scores measured by the PGSAS‐45 were comparable between the two groups (*p* > 0.05; Figure S2).

## 4 DISSUSION

To date, TG remains the preferred surgical approach for patients with early proximal gastric cancer unsuitable for endoscopic treatment [27]. With advancing understanding of lymphatic metastasis patterns and the development of anti‐reflux reconstruction techniques, PG has been increasingly adopted in clinical practice [28, 29]. In addition to PG‐DTR, other reconstruction methods such as Kamikawa, SOFY, and tubular stomach reconstruction have been proposed to address postgastrectomy reflux and functional preservation. While Kamikawa and SOFY focus on creating anti‐reflux valves through flap or fundoplication techniques, tubular reconstruction aims to preserve gastric reservoir function by shaping a narrow gastric tube [30, 31]. Although these techniques aim to optimize outcomes, no consensus exists on the optimal approach due to variations in technical complexity, postoperative complications, and nutritional outcomes.

PG‐DTR has been established as a primary surgical approach for early‐stage proximal gastric cancer in our institution, supported by its endorsement in the 2020 Chinese Consensus on Digestive Tract Reconstruction (evidence level II C, recommendation grade B) [32] and by its dual‐pathway mechanism, which optimizes both anti‐reflux efficacy and nutrient absorption.

In this study, the majority of PG‐DTR patients underwent postoperative fluoroscopy, showing contrast agent passage through both the remnant stomach and jejunum into the distal jejunum. However, in two cases, the contrast agent passed only through the jejunum, and in one case, only through the remnant stomach. When most food bypasses the remnant stomach via the jejunal pathway, the benefits of PG may diminish, resembling those of TG [33]. A larger jejunogastrostomy anastomosis reduces resistance to food entry into the remnant stomach, allowing greater food volume [34]. In line with previous recommendations [35, 36], this study used a 60 mm linear stapler to expand the jejunogastrostomy anastomosis.

Although PG‐DTR involves more surgical steps and an additional anastomosis compared to TG‐RY, advancements in techniques and instrumentation prevented any increase in operation time or anastomotic leakage rates. Furthermore, the two procedures showed no significant differences in blood loss, radical resection rates, intraoperative blood transfusion, time to bowel function recovery, POCs, or mortality. These results are consistent with previous studies [16, 37, 38], supporting the satisfactory outcomes, feasibility, and safety of PG‐DTR.

Since TG entails a more extensive lymphadenectomy that includes stations #4d, #5, #6, and #12a—stations not routinely dissected in PG per the Japanese Gastric Cancer Treatment Guidelines [26]—the TG‐RY group had more dissected lymph nodes in this study. This difference was entirely attributable to the retrieval of lymph nodes from these additional stations. Importantly, none of these additionally retrieved lymph nodes (#4d, #5, #6, and #12a) in the TG‐RY group were positive for metastasis. Oncological safety after PG depends not only on the extent of lymph node dissection but also on the metastatic risk in distal nodal basins. Our findings align with previous reports indicating a very low metastatic rate in stations #4d, #5, #6, and #12a—even zero among T2 tumors—which supports the oncological feasibility of omitting their dissection during PG [39]. A multicenter retrospective study in Japan found that for EGJ carcinoma ≤ 4 cm in diameter, distal gastric lymph node metastasis is rare, supporting the feasibility of transabdominal PG [40]. In this study, OS and DFS were comparable between the PG‐DTR and TG‐RY groups. For early‐stage gastric cancer, PG effectively ensures oncological safety by addressing necessary lymph node dissection while minimizing resection extent [41, 42, 43].

Postoperative serum iron levels did not differ significantly between the PG‐DTR and TG‐RY groups. When serum iron or ferritin levels fall below normal, our institution routinely administers oral iron supplementation. However, hemoglobin levels were higher in the PG‐DTR group. The preservation of a portion of the stomach in PG‐DTR may permit limited production of gastric acid and intrinsic factor, potentially enhancing the absorption of iron and vitamin B12, thereby supporting hemoglobin maintenance. Additionally, the DTR approach promotes more efficient nutrient absorption, which may further contribute to improved hemoglobin levels [44, 45, 46, 47].

One of the benefits of TG for proximal gastric cancer is its lower incidence of reflux esophagitis [48, 49]. In this study, the DTR approach effectively reduced reflux symptoms to a level comparable to TG. The interposed jejunal segment between the remnant stomach and esophagus increases the distance for potential reflux while also allowing partial food passage through the jejunum, thereby alleviating food stasis in the remnant stomach [15].

Although a small proportion of patients did not complete QOL assessments at 6 and 12 months postoperatively, the study employed a LMM, which is well‐suited for longitudinal data analysis and can accommodate missing data under the assumption that data are missing at random (MAR) [50]. This approach allowed for a robust comparison between the TG‐RY and PG‐DTR groups. QOL scores were comparable between the two groups, except for a greater decrease in body weight observed in the TG‐RY group during postoperative follow‐up. Park et al. [13] used the European Organization for Research and Treatment of Cancer (EORTC) core questionnaires (QLQ‐C30) and the gastric cancer‐specific module (QLQ‐STO22) to assess postoperative QOL in PG and TG, finding similar outcomes between the groups up to 2 years after surgery. Another study found no clinically significant difference in food‐related symptoms or QOL between PG and TG [51]. However, Sato et al. [52] found that PG‐DT resulted in better QOL outcomes, particularly in diarrhea symptoms and food intake quality scores. Similarly, another study indicated that patients undergoing PG‐DT had improved QOL, with reduced reflux esophagitis and higher scores in global health and emotional functioning, along with fewer symptoms such as nausea, vomiting, and pain [53]. The variations in findings on PG‐DT's impact on QOL may stem from differences in assessment tools, as some scales emphasize gastrointestinal symptoms more than others, as well as from patient characteristics and surgical expertise across studies.

Nutritional indicators, including body weight, hemoglobin, albumin, and total protein, consistently remained higher in the PG‐DTR group, suggesting that DTR may improve postoperative nutritional status. Notably, total protein and albumin reached their lowest levels, and the most significant weight loss occurred at 3 months postoperatively. This period represents a critical phase in recovery, as highlighted by multiple studies emphasizing the importance of nutritional status at 3 months post‐surgery [54, 55]. Accordingly, we used GLIM to assess malnutrition at this specific time point and identified TG‐RY as an independent risk factor for postoperative malnutrition. The preservation of part of the stomach and the open duodenal pathway in PG‐DTR promotes duodenal functionality, stimulating the secretion of cholecystokinin and secretin, which aid in food digestion and absorption. Additionally, retaining part of the stomach preserves its grinding and digestive functions, facilitating more effective bile and food transport and mixing, which supports overall nutritional maintenance [14, 44, 56, 57].

This study has several limitations. First, although recent findings indicate that PG does not compromise OS in stage III gastric cancer [58], our study included only cases of stage cT1 to cT2. Second, differences in postoperative serum vitamin B12 levels between the two groups may partially explain the lower hemoglobin levels observed in the TG‐RY group [57, 59]. Our study did not measure vitamin B12 levels or track the number of patients taking vitamin B12 supplements. Third, as a non‐randomized study, selection bias may exist despite PSM, as treatment allocation was based on clinical and anatomical criteria rather than randomization. Finally, although a small number of patients were excluded based on postoperative fluoroscopy findings (exclusive passage through either the remnant stomach or the jejunal limb), this criterion was used to ensure functional validation of the double‐tract physiology and maintain cohort homogeneity.

## 5 Conclusion

In summary, PG‐DTR demonstrated significant advantages over TG‐RY in enhancing postoperative nutritional status for patients with proximal gastric cancer, while maintaining comparable short‐term outcomes and mid‐term prognosis, establishing it as a viable surgical option. Further long‐term studies are warranted to more precisely define these benefits and to optimize patient outcomes.

## Author Contributions

**Ying Liu:** conceptualization (equal), data curation (equal), methodology (equal), project administration (lead), resources (lead), writing – original draft (equal). **Mingfang Yan:** data curation (lead), writing – original draft (lead), formal analysis (equal), funding acquisition (equal), resources (equal). **Zhaoyan Lin:** conceptualization (equal), methodology (lead), writing – review and editing (lead), formal analysis (lead). **Shenghong Wei:** data curation (equal), writing – review and editing (equal). **Yangming Li:** data curation (equal), methodology (equal). **Zhenmeng Lin:** data curation (equal), writing – review and editing (equal), funding acquisition (lead). **Xingfa Chen:** conceptualization (lead), data curation (equal), software (lead), writing – review and editing (equal).

## Ethics Statement

The study was conducted according to the Declaration of Helsinki, and approved by the Ethics Committee of Fujian Cancer Hospital (SQ2021‐025‐01). All patients provided written informed consent and agreed to participate in data collection.

## Conflicts of Interest

The authors declare no conflicts of interest.

## Supporting information


**Figures S1–S2:** cam471258‐sup‐0001‐FigureS1‐S2.doc.

## Data Availability

The original data can be obtained from the corresponding author upon reasonable request.

## References

[1] H. Sung , J. Ferlay , R. L. Siegel , et al., “Global Cancer Statistics 2020: GLOBOCAN Estimates of Incidence and Mortality Worldwide for 36 Cancers in 185 Countries,” CA: A Cancer Journal for Clinicians 71, no. 3 (2021): 209–249. 33538338 10.3322/caac.21660

[2] M. Arnold , J. Y. Park , M. C. Camargo , N. Lunet , D. Forman , and I. Soerjomataram , “Is Gastric Cancer Becoming a Rare Disease? A Global Assessment of Predicted Incidence Trends to 2035,” Gut 69, no. 5 (2020): 823–829. 32001553 10.1136/gutjnl-2019-320234 PMC8520492

[3] N. Manabe , K. Matsueda , and K. Haruma , “Epidemiological Review of Gastroesophageal Junction Adenocarcinoma in Asian Countries,” Digestion 103, no. 1 (2022): 29–36. 34718236 10.1159/000519602

[4] Y. Huang , Y. Shao , X. Yu , C. Chen , J. Guo , and G. Ye , “Global Progress and Future Prospects of Early Gastric Cancer Screening,” Journal of Cancer 15, no. 10 (2024): 3045–3064. 38706913 10.7150/jca.95311 PMC11064266

[5] C. B. Conti , S. Agnesi , M. Scaravaglio , et al., “Early Gastric Cancer: Update on Prevention, Diagnosis and Treatment,” International Journal of Environmental Research and Public Health 20, no. 3 (2023): 2149. 36767516 10.3390/ijerph20032149 PMC9916026

[6] J. Yu , Z. Wang , H. Yang , et al., “Long‐Term Health‐Related Quality of Life in Patients With Gastric Cancer After Total or Distal Gastrectomy: A Propensity Score‐Matched Cohort Study,” International Journal of Surgery 109, no. 11 (2023): 3283–3293. 37526103 10.1097/JS9.0000000000000620 PMC10651271

[7] S. S. Lee , H. Y. Chung , O. K. Kwon , and W. Yu , “Long‐Term Quality of Life After Distal Subtotal and Total Gastrectomy: Symptom‐ and Behavior‐Oriented Consequences,” Annals of Surgery 263, no. 4 (2016): 738–744. 26501699 10.1097/SLA.0000000000001481

[8] Japanese Gastric Cancer Association , “Japanese Gastric Cancer Treatment Guidelines 2018 (5th Edition),” Gastric Cancer 24, no. 1 (2021): 1–21. 32060757 10.1007/s10120-020-01042-y PMC7790804

[9] S. Nunobe and S. Ida , “Current Status of Proximal Gastrectomy for Gastric and Esophagogastric Junctional Cancer: A Review,” Annals of Gastroenterological Surgery 4, no. 5 (2020): 498–504. 33005844 10.1002/ags3.12365 PMC7511558

[10] L. Li , X. Cai , Z. Liu , Y. Mou , and Y. Wang , “Digestive Tract Reconstruction After Laparoscopic Proximal Gastrectomy for Gastric Cancer: A Systematic Review,” Journal of Cancer 14, no. 16 (2023): 3139–3150. 37859825 10.7150/jca.87315 PMC10583589

[11] K. V. Stegniy , E. V. Maslyantsev , R. A. Goncharuk , A. A. Krekoten , T. A. Kulakova , and E. R. Dvoinikova , “Double‐Tract Reconstruction for Oesofagocardial Gastric Cancer: A Systematic Review,” Annals of Medicine and Surgery 67 (2021): 102496. 34194733 10.1016/j.amsu.2021.102496 PMC8226393

[12] T. S. Lewis and Y. Feng , “A Review on Double Tract Reconstruction After Proximal Gastrectomy for Proximal Gastric Cancer,” Annals of Medicine and Surgery 79 (2022): 103879. 35860159 10.1016/j.amsu.2022.103879 PMC9289219

[13] J. Y. Park , K. B. Park , O. K. Kwon , and W. Yu , “Comparison of Laparoscopic Proximal Gastrectomy With Double‐Tract Reconstruction and Laparoscopic Total Gastrectomy in Terms of Nutritional Status or Quality of Life in Early Gastric Cancer Patients,” European Journal of Surgical Oncology 44, no. 12 (2018): 1963–1970. 30197164 10.1016/j.ejso.2018.08.014

[14] K. Ying , W. Bai , G. Yan , Z. Xu , S. Du , and C. Dang , “The Comparison of Long‐Term Oncological Outcomes and Complications After Proximal Gastrectomy With Double Tract Reconstruction Versus Total Gastrectomy for Proximal Gastric Cancer,” World Journal of Surgical Oncology 21, no. 1 (2023): 101. 36949503 10.1186/s12957-023-02985-z PMC10035210

[15] H. J. Ko , K. H. Kim , S. H. Lee , et al., “Can Proximal Gastrectomy With Double‐Tract Reconstruction Replace Total Gastrectomy? A Propensity Score Matching Analysis,” Journal of Gastrointestinal Surgery 24, no. 3 (2020): 516–524. 30937710 10.1007/s11605-019-04195-z

[16] S. Li , L. Gu , Z. Shen , D. Mao , P. A. Khadaroo , and H. Su , “A Meta‐Analysis of Comparison of Proximal Gastrectomy With Double‐Tract Reconstruction and Total Gastrectomy for Proximal Early Gastric Cancer,” BMC Surgery 19, no. 1 (2019): 117. 31438918 10.1186/s12893-019-0584-7 PMC6704512

[17] K. Uchiyama , T. Ando , E. Kishimoto , et al., “Correlation of Gastrointestinal Symptom Rating Scale and Frequency Scale for the Symptoms of Gastroesophageal Reflux Disease With Endoscopic Findings,” Scandinavian Journal of Gastroenterology 59 (2024): 1–9. 39301940 10.1080/00365521.2024.2406537

[18] N. Ikoma , M. Blum , J. S. Estrella , et al., “Evaluation of the American Joint Committee on Cancer 8th Edition Staging System for Gastric Cancer Patients After Preoperative Therapy,” Gastric Cancer 21, no. 1 (2018): 74–83. 28643144 10.1007/s10120-017-0743-4 PMC7703858

[19] X. You , Q. Zhou , J. Song , L. Gan , J. Chen , and H. Shen , “Preoperative Albumin‐To‐Fibrinogen Ratio Predicts Severe Postoperative Complications in Elderly Gastric Cancer Subjects After Radical Laparoscopic Gastrectomy,” BMC Cancer 19, no. 1 (2019): 931. 31533682 10.1186/s12885-019-6143-x PMC6751606

[20] Z. Lin , M. Yan , Z. Lin , et al., “Short‐Term Outcomes of Distal Gastrectomy Versus Total Gastrectomy for Gastric Cancer Under Enhanced Recovery After Surgery: A Propensity Score‐Matched Analysis,” Scientific Reports 14, no. 1 (2024): 17594. 39080478 10.1038/s41598-024-68787-9 PMC11289314

[21] S. W. Lee , M. Kaji , Y. Uenosono , et al., “The Evaluation of the Postoperative Quality of Life in Patients Undergoing Radical Gastrectomy for Esophagogastric Junction Cancer Using the Postgastrectomy Syndrome Assessment Scale‐45: A Nationwide Multi‐Institutional Study,” Surgery Today 52, no. 5 (2022): 832–843. 34734320 10.1007/s00595-021-02400-8

[22] S. Nunobe , M. Takahashi , S. Kinami , et al., “Evaluation of Postgastrectomy Symptoms and Daily Lives of Small Remnant Distal Gastrectomy for Upper‐Third Gastric Cancer Using a Large‐Scale Questionnaire Survey,” Annals of Gastroenterological Surgery 6, no. 3 (2022): 355–365. 35634182 10.1002/ags3.12536 PMC9130885

[23] T. Tsumura , S. Kuroda , M. Nishizaki , et al., “Short‐Term and Long‐Term Comparisons of Laparoscopy‐Assisted Proximal Gastrectomy With Esophagogastrostomy by the Double‐Flap Technique and Laparoscopy‐Assisted Total Gastrectomy for Proximal Gastric Cancer,” PLoS One 15, no. 11 (2020): e0242223. 33180871 10.1371/journal.pone.0242223 PMC7660475

[24] H. L. Zheng , J. Lin , L. L. Shen , et al., “The GLIM Criteria as an Effective Tool for Survival Prediction in Gastric Cancer Patients,” European Journal of Surgical Oncology 49, no. 5 (2023): 964–973. 36958948 10.1016/j.ejso.2023.01.009

[25] W. Huang , C. Wang , Y. Wang , et al., “Predicting Malnutrition in Gastric Cancer Patients Using Computed Tomography (CT) Deep Learning Features and Clinical Data,” Clinical Nutrition 43, no. 3 (2024): 881–891. 38377634 10.1016/j.clnu.2024.02.005

[26] Japanese Gastric Cancer Association , “Japanese Gastric Cancer Treatment Guidelines 2021 (6th Edition),” Gastric Cancer 26, no. 1 (2023): 1–25. 36342574 10.1007/s10120-022-01331-8 PMC9813208

[27] H. Yamashita , K. Toyota , C. Kunisaki , et al., “Current Status of Gastrectomy and Reconstruction Types for Patients With Proximal Gastric Cancer in Japan,” Asian Journal of Surgery 46, no. 10 (2023): 4344–4351. 36464591 10.1016/j.asjsur.2022.11.069

[28] K. K. Sun and Y. Y. Wu , “Current Status of Laparoscopic Proximal Gastrectomy in Proximal Gastric Cancer: Technical Details and Oncologic Outcomes,” Asian Journal of Surgery 44, no. 1 (2021): 54–58. 32981822 10.1016/j.asjsur.2020.09.006

[29] Y. Kano , M. Ohashi , and S. Nunobe , “Laparoscopic Function‐Preserving Gastrectomy for Proximal Gastric Cancer or Esophagogastric Junction Cancer: A Narrative Review,” Cancers 15, no. 1 (2023): 311. 36612308 10.3390/cancers15010311 PMC9818997

[30] C. Y. Wu , J. A. Lin , and K. Ye , “Clinical Efficacy of Modified Kamikawa Anastomosis in Patients With Laparoscopic Proximal Gastrectomy,” World Journal of Gastroenterology 16, no. 1 (2024): 113–123. 10.4240/wjgs.v16.i1.113 PMC10845268 38328314

[31] Y. Yamashita , T. Tatsubayashi , K. Okumura , T. Miyamoto , and K. Ueno , “Modified Side Overlap Esophagogastrostomy After Laparoscopic Proximal Gastrectomy,” Annals of Gastroenterological Surgery 6, no. 4 (2022): 594–599. 35847432 10.1002/ags3.12549 PMC9271030

[32] Chinese Gastric Cancer Association , “Chinese Consensus on Digestive Tract Reconstruction After Proximal Gastrectomy (2024 Edition),” Zhonghua Wei Chang Wai Ke Za Zhi 27, no. 10 (2024): 983–995. 39428219 10.3760/cma.j.cn441530-20240918-00323

[33] K. Tanaka , Y. Ebihara , Y. Kurashima , et al., “Laparoscopic Proximal Gastrectomy With Oblique Jejunogastrostomy,” Langenbeck's Archives of Surgery 402, no. 6 (2017): 995–1002. 10.1007/s00423-017-1587-4 28493146

[34] S. Kamiya , T. Namikawa , M. Takahashi , et al., “Optimal Procedures for Double Tract Reconstruction After Proximal Gastrectomy Assessed by Postgastrectomy Syndrome Assessment Scale‐45,” Journal of Gastrointestinal Surgery 26, no. 9 (2022): 1817–1829. 35524078 10.1007/s11605-022-05328-7

[35] D. Fujimoto , K. Taniguchi , and H. Kobayashi , “Double‐Tract Reconstruction Designed to Allow More Food Flow to the Remnant Stomach After Laparoscopic Proximal Gastrectomy,” World Journal of Surgery 44, no. 8 (2020): 2728–2735. 32236727 10.1007/s00268-020-05496-0

[36] J. Hong , L. Qian , Y. P. Wang , J. Wang , L. C. Hua , and H. K. Hao , “A Novel Method of Delta‐Shaped Intracorporeal Double‐Tract Reconstruction in Totally Laparoscopic Proximal Gastrectomy,” Surgical Endoscopy 30, no. 6 (2016): 2396–2403. 26416371 10.1007/s00464-015-4490-5

[37] J. Hong , S. Y. Wang , and H. K. Hao , “A Comparative Study of Double‐Tract Reconstruction and Roux‐En‐Y After Gastrectomy for Gastric Cancer,” Surgical Laparoscopy, Endoscopy & Percutaneous Techniques 29, no. 2 (2019): 82–89. 10.1097/SLE.0000000000000639 30720693

[38] Z. Li , J. Dong , and Q. Huang , “Feasibility of Laparoscopic Proximal Gastrectomy With Piggyback Jejunal Interposition Double‐Tract Reconstruction for Proximal Gastric Cancer: A Propensity Score‐Matching Analysis,” Journal of Minimal Access Surgery 19, no. 1 (2023): 20–27. 36722527 10.4103/jmas.jmas_46_22 PMC10034807

[39] M. Yura , T. Yoshikawa , S. Otsuki , et al., “Oncological Safety of Proximal Gastrectomy for T2/T3 Proximal Gastric Cancer,” Gastric Cancer 22, no. 5 (2019): 1029–1035. 30778799 10.1007/s10120-019-00938-8

[40] H. Yamashita , Y. Seto , T. Sano , H. Makuuchi , N. Ando , and M. Sasako , “Results of a Nation‐Wide Retrospective Study of Lymphadenectomy for Esophagogastric Junction Carcinoma,” Gastric Cancer 20, no. Suppl 1 (2017): 69–83. 27796514 10.1007/s10120-016-0663-8

[41] J. W. Xiao , Z. L. Liu , P. C. Ye , et al., “Clinical Comparison of Antrum‐Preserving Double Tract Reconstruction vs Roux‐En‐Y Reconstruction After Gastrectomy for Siewert Types II and III Adenocarcinoma of the Esophagogastric Junction,” World Journal of Gastroenterology 21, no. 34 (2015): 9999–10007. 26379405 10.3748/wjg.v21.i34.9999 PMC4566393

[42] F. Ma , D. Guo , B. Zhang , et al., “Short and Long‐Term Outcomes After Proximal Gastrectomy With Double Tract Reconstruction for Siewert Type III Adenocarcinoma of the Esophagogastric Junction: A Propensity Score Matching Study From a 10‐Year Experience in a High‐Volume Hospital,” Journal of Gastrointestinal Oncology 11, no. 6 (2020): 1261–1273. 33456999 10.21037/jgo-20-475 PMC7807272

[43] W. Dai , F. Wen , X. Li , and Z. Fu , “The Long‐Term Results of Proximal Gastrectomy for Proximal Gastric Cancer: A Propensity Score Matching Analysis Based on SEER Database,” American Surgeon 90, no. 11 (2024): 3015–3023. 38867409 10.1177/00031348241260273

[44] K. Kimura , Y. Ebihara , K. Tanaka , et al., “Initial Results of Laparoscopic Proximal Gastrectomy With Double‐Tract Reconstruction Using Oblique Jejunogastrostomy Method on the Long‐Term Outcome of Postoperative Nutritional Status: A Propensity Score‐Matched Study,” Surgical Laparoscopy, Endoscopy & Percutaneous Techniques 31, no. 5 (2021): 603–607. 10.1097/SLE.0000000000000954 34049369

[45] Z. Xu , W. Lin , S. Yan , et al., “The Short‐Term and Long‐Term Outcomes of Laparoscopy‐Assisted Proximal Gastrectomy With Double‐Tract Reconstruction Versus Laparoscopy‐Assisted Total Gastrectomy With Roux‐En‐Y Reconstruction for Adenocarcinoma of the Esophagogastric Junction: A Multicenter Study Based on Propensity Score Matching Analysis,” Gastroenterology Research and Practice 2024 (2024): 5517459. 38882392 10.1155/2024/5517459 PMC11178406

[46] G. Zhu , X. Jiao , S. Zhou , et al., “Can Proximal Gastrectomy With Double‐Tract Reconstruction Replace Total Gastrectomy? A Meta‐Analysis of Randomized Controlled Trials and Propensity Score‐Matched Studies,” BMC Gastroenterology 24, no. 1 (2024): 230. 39044132 10.1186/s12876-024-03323-7 PMC11267959

[47] J. H. Song , S. H. Park , M. Cho , Y. M. Kim , W. J. Hyung , and H. I. Kim , “Proximal Gastrectomy Is Associated With Lower Incidence of Anemia and Vitamin B12 Deficiency Compared to Total Gastrectomy in Patients With Upper Gastric Cancer,” Cancer Research and Treatment 175 (2024): 174–185. 10.4143/crt.2024.319 PMC11729319 38965924

[48] M. Yamasaki , S. Takiguchi , T. Omori , et al., “Multicenter Prospective Trial of Total Gastrectomy Versus Proximal Gastrectomy for Upper Third cT1 Gastric Cancer,” Gastric Cancer 24, no. 2 (2021): 535–543. 33118118 10.1007/s10120-020-01129-6

[49] Y. W. Pu , W. Gong , Y. Y. Wu , Q. Chen , T. F. He , and C. G. Xing , “Proximal Gastrectomy Versus Total Gastrectomy for Proximal Gastric Carcinoma. A Meta‐Analysis on Postoperative Complications, 5‐Year Survival, and Recurrence Rate,” Saudi Medical Journal 34, no. 12 (2013): 1223–1228. 24343461

[50] M. C. Ard , N. Raghavan , and S. D. Edland , “Optimal Composite Scores for Longitudinal Clinical Trials Under the Linear Mixed Effects Model,” Pharmaceutical Statistics 14, no. 5 (2015): 418–426. 26223663 10.1002/pst.1701 PMC5132034

[51] A. Irfan , T. Yang , M. Bowring , A. B. Blair , and M. Duncan , “Proximal vs. Total Gastrectomy: Is There a Difference in Quality of Life for Patients,” American Surgeon 89, no. 3 (2023): 401–406. 35448929 10.1177/00031348211029850

[52] R. Sato , T. Kinoshita , E. Akimoto , M. Yoshida , Y. Nishiguchi , and J. Harada , “Feasibility and Quality of Life Assessment of Laparoscopic Proximal Gastrectomy Using Double‐Tract Reconstruction,” Langenbeck's Archives of Surgery 406, no. 2 (2021): 479–489. 10.1007/s00423-020-02076-7 33452650

[53] J. Chen , F. Wang , S. Gao , et al., “Surgical Outcomes of Laparoscopic Proximal Gastrectomy for Upper‐Third Gastric Cancer: Esophagogastrostomy, Gastric Tube Reconstruction, and Double‐Tract Reconstruction,” BMC Surgery 23, no. 1 (2023): 309. 37828530 10.1186/s12893-023-02219-9 PMC10571476

[54] T. Dai , D. Wu , J. Tang , Z. Liu , and M. Zhang , “Construction and Validation of a Predictive Model for the Risk of Three‐Month‐Postoperative Malnutrition in Patients With Gastric Cancer: A Retrospective Case‐Control Study,” Journal of Gastrointestinal Oncology 14, no. 1 (2023): 128–145. 36915453 10.21037/jgo-22-1307 PMC10007955

[55] Q. Tian , L. Qin , W. Zhu , S. Xiong , and B. Wu , “Analysis of Factors Contributing to Postoperative Body Weight Change in Patients With Gastric Cancer: Based on Generalized Estimation Equation,” PeerJ 8 (2020): e9390. 32728490 10.7717/peerj.9390 PMC7357557

[56] L. Wang , Y. Xia , T. Jiang , et al., “Short‐Term Surgical Outcomes of Laparoscopic Proximal Gastrectomy With Double‐Tract Reconstruction Versus Laparoscopic Total Gastrectomy for Adenocarcinoma of Esophagogastric Junction: A Matched‐Cohort Study,” Journal of Surgical Research 246 (2020): 292–299. 31630013 10.1016/j.jss.2019.09.022

[57] R. Xiang , W. Song , J. Ren , W. Lu , H. Zhang , and T. Fu , “Proximal Gastrectomy With Double‐Tract Reconstruction Versus Total Gastrectomy for Proximal Early Gastric Cancer: A Systematic Review and Meta‐Analysis,” Medicine (Baltimore) 100, no. 45 (2021): e27818. 34766595 10.1097/MD.0000000000027818 PMC8589236

[58] T. K. Uprak , M. Ergenç , A. Akmercan , and C. Yeğen , “Outcomes of Proximal Versus Total Gastrectomy for Proximal Gastric Cancer: A Propensity Score‐Matched Analysis of a Western Center Experience,” Journal of Gastrointestinal Surgery 27, no. 8 (2023): 1560–1567. 37130980 10.1007/s11605-023-05686-w

[59] Y. Wang , K. Chen , X. Feng , et al., “Comparison of Laparoscopic Proximal Gastrectomy With Double‐Tract Reconstruction and Laparoscopic Total Gastrectomy for Proximal Gastric Cancer With Stage cT1‐2,” Medicine 100, no. 51 (2021): e28115. 34941055 10.1097/MD.0000000000028115 PMC8702284

