# Supplementary material for: Short‐ and Mid‐Term Outcomes of Proximal Gastrectomy With Double‐Tract Reconstruction Versus Total Gastrectomy in Early‐Stage Proximal Gastric Cancer
Source: Cancer Med. 2025 Sep 17;14(18):e71258. doi: 10.1002/cam4.71258 (PMC12441810; doi:10.1002/cam4.71258)
Supplement: Supplementary file 1 — Figures S1–S2: cam471258‐sup‐0001‐FigureS1‐S2.doc. [file CAM4-14-e71258-s001.doc]

**(A)
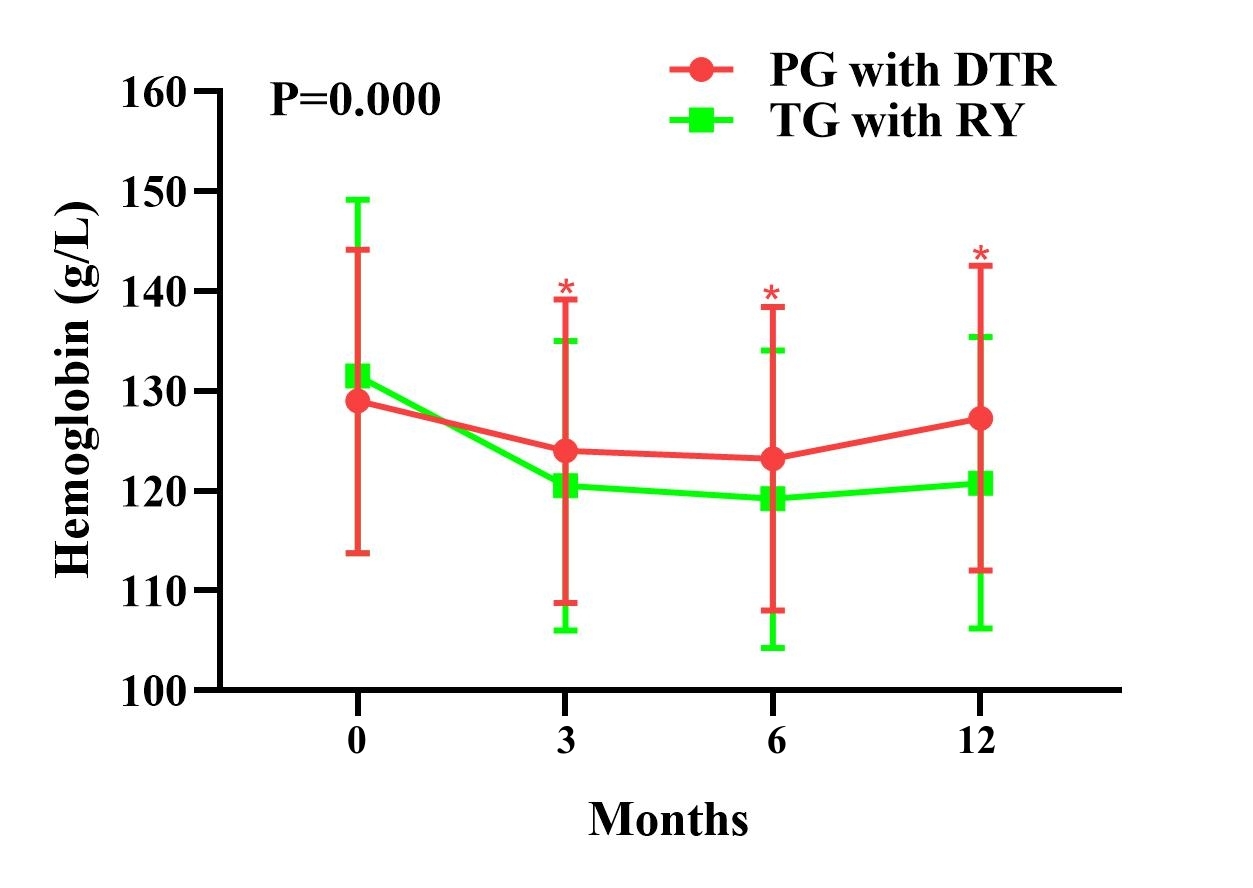
(B)
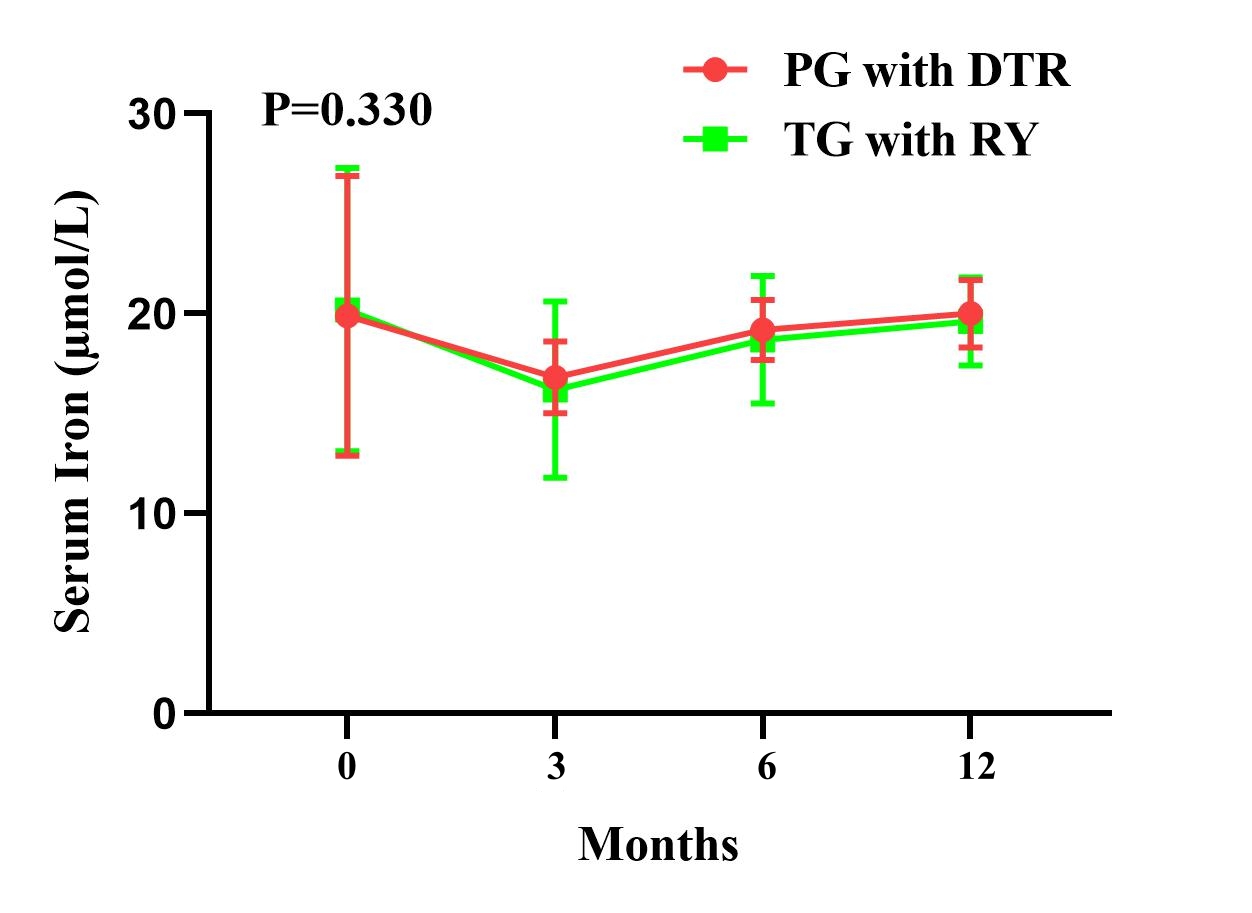
**

**(C)
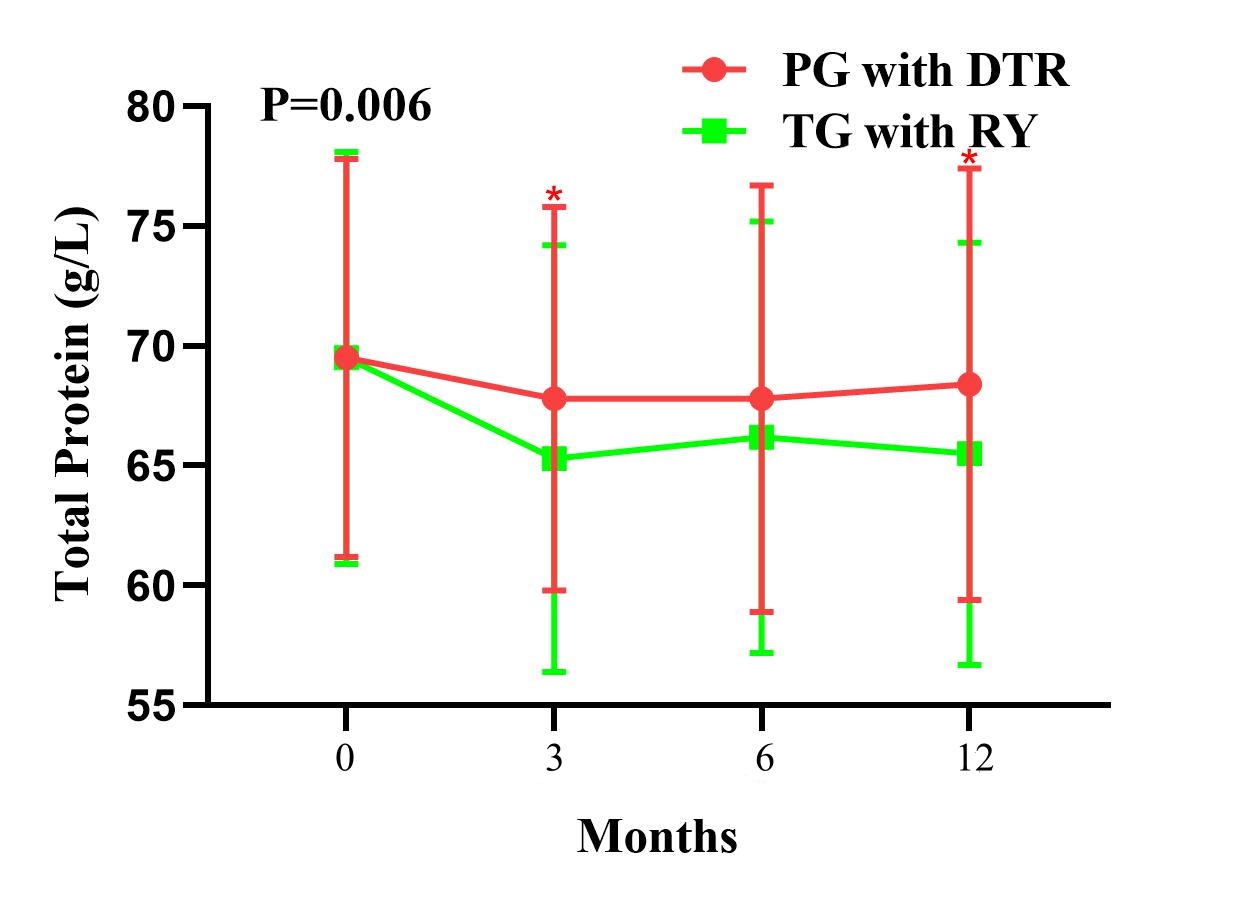
 (D)
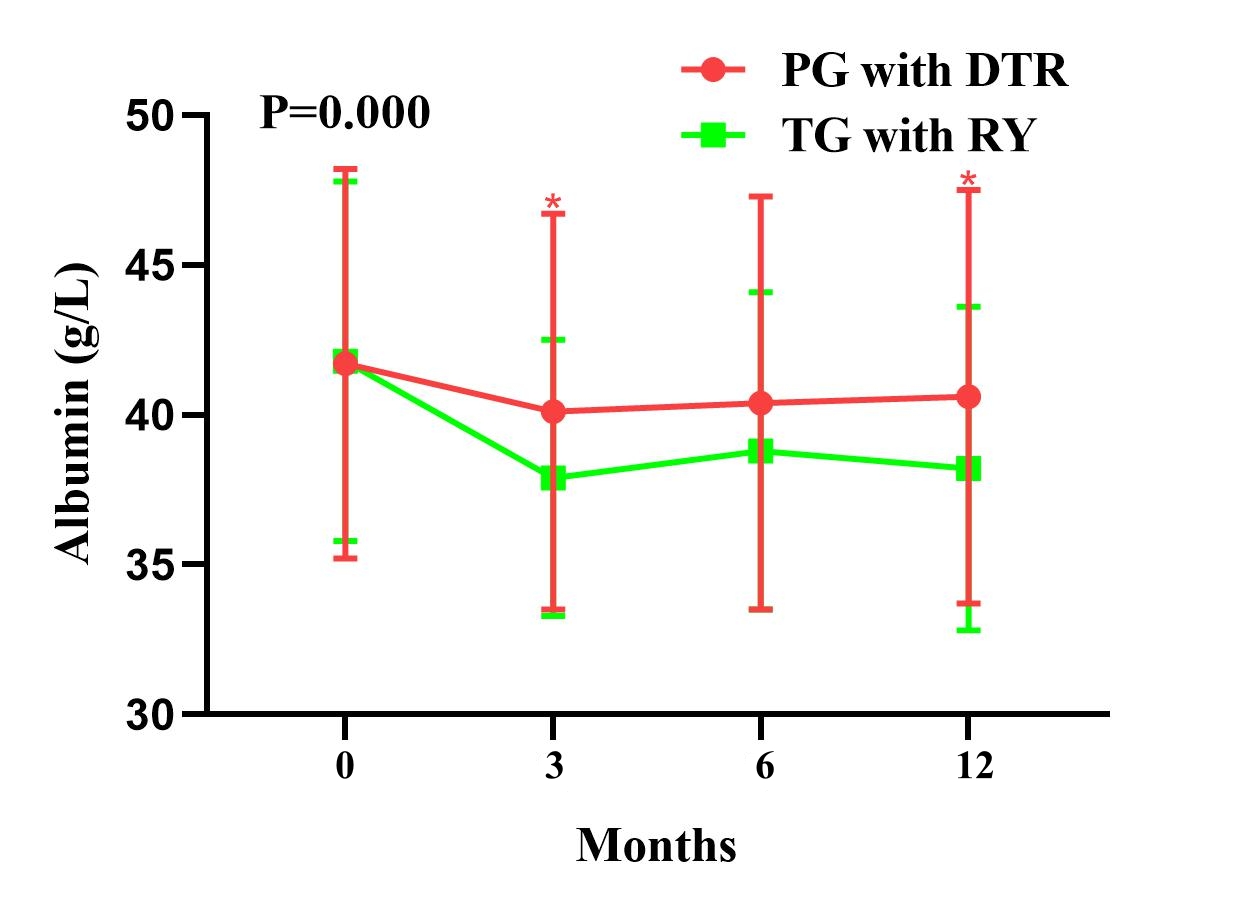
**

SUPPLEMENTARY FIGURES S1 Preoperative and postoperative levels of hematologic parameters. (A) Hemoglobin; (B) serum iron; (C) total protein; (D) serum albumin.

*Significant difference with P <0.05.

**(A)
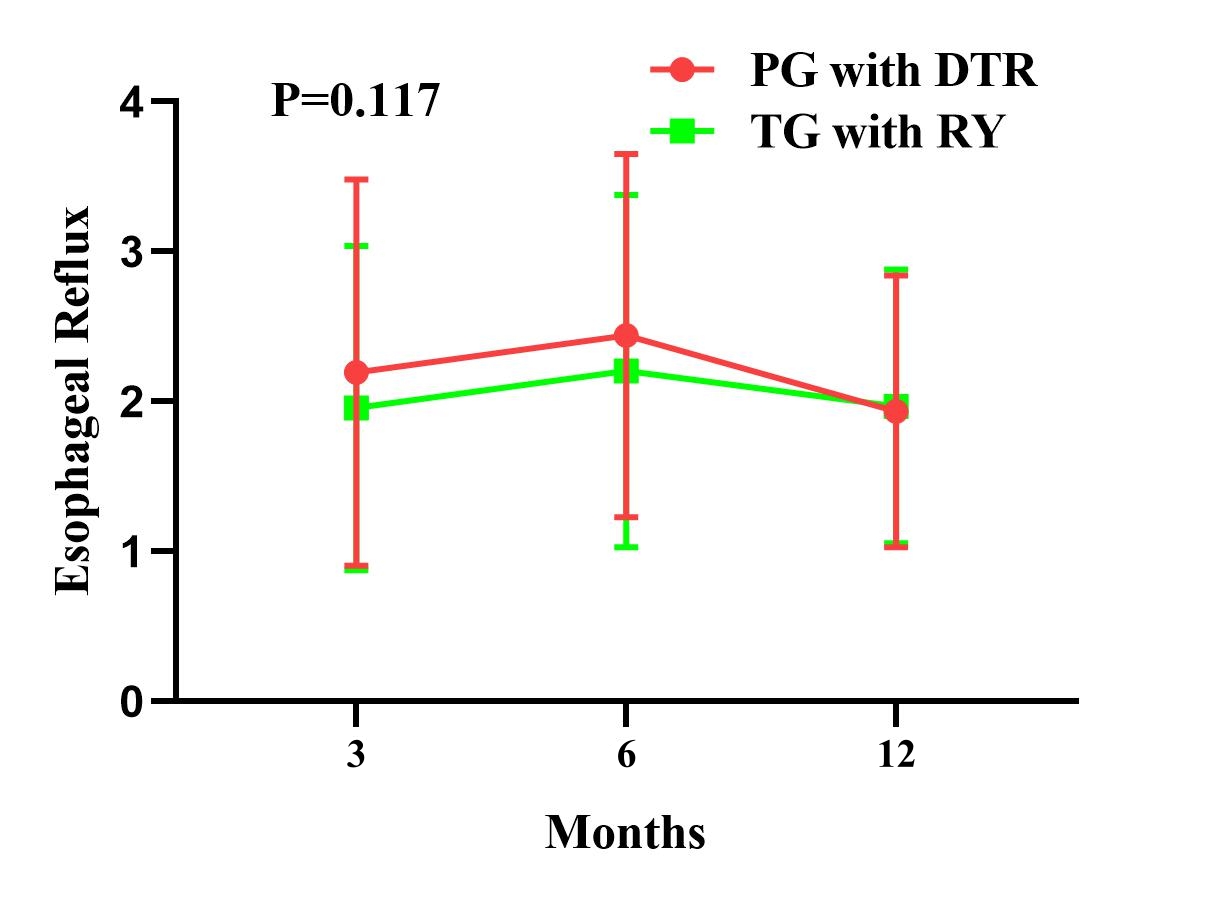
(B)
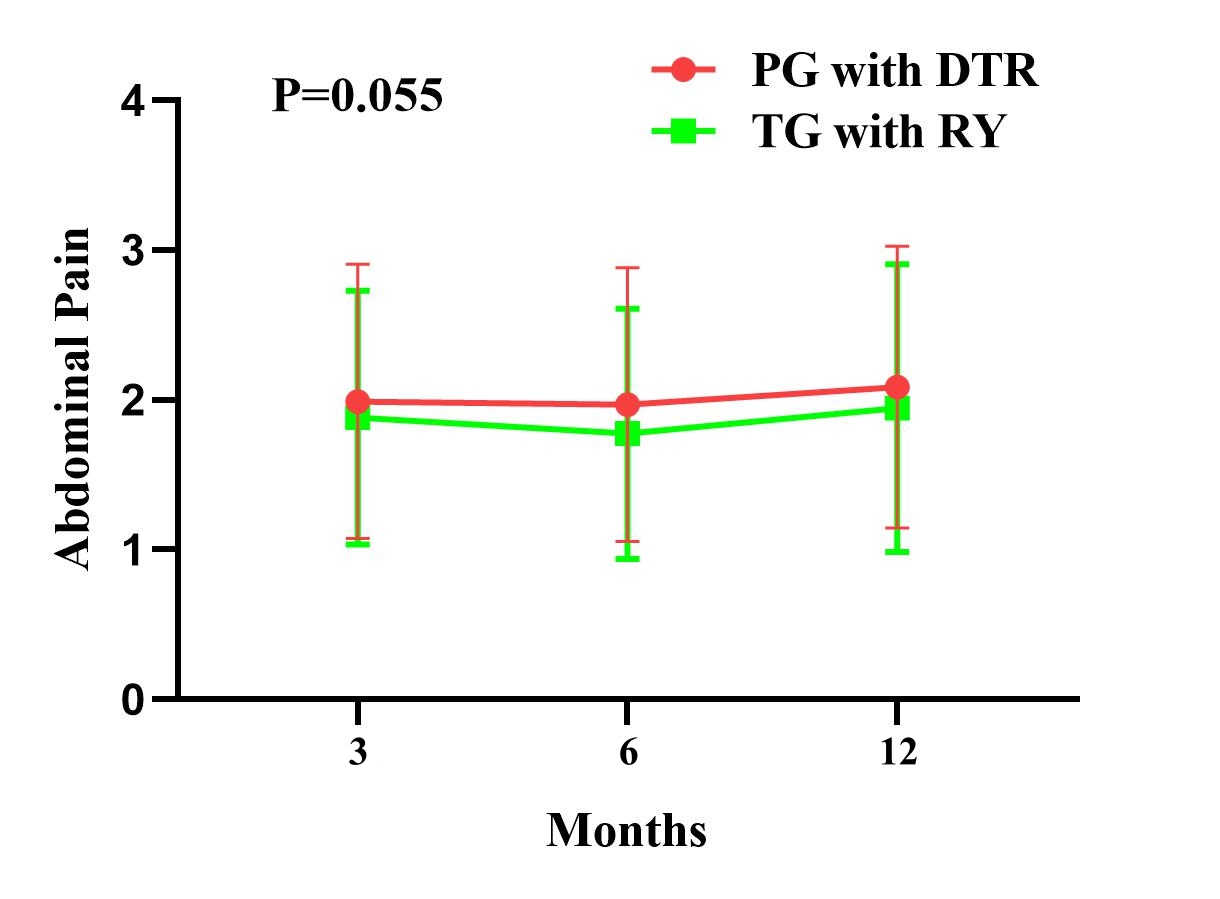
**

**(C)
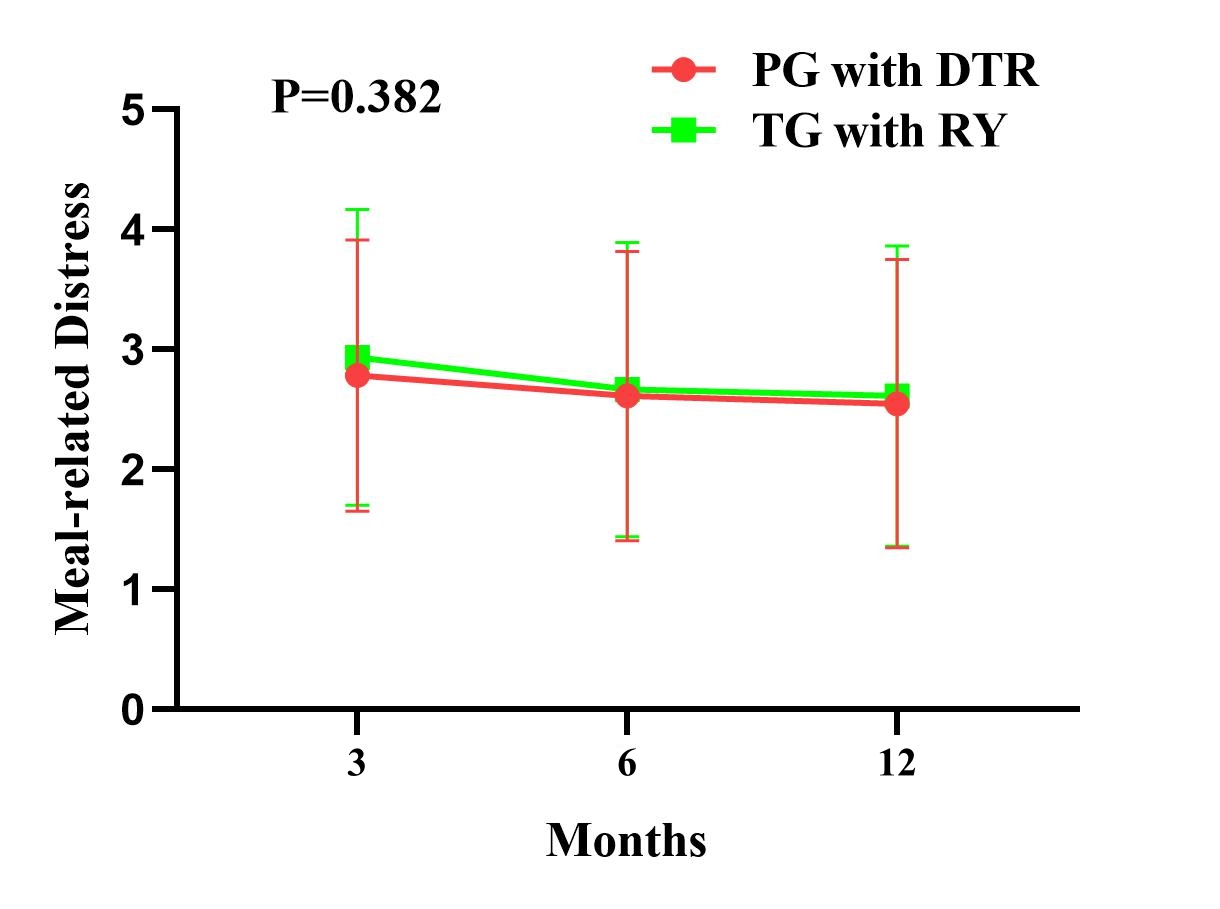
(D)
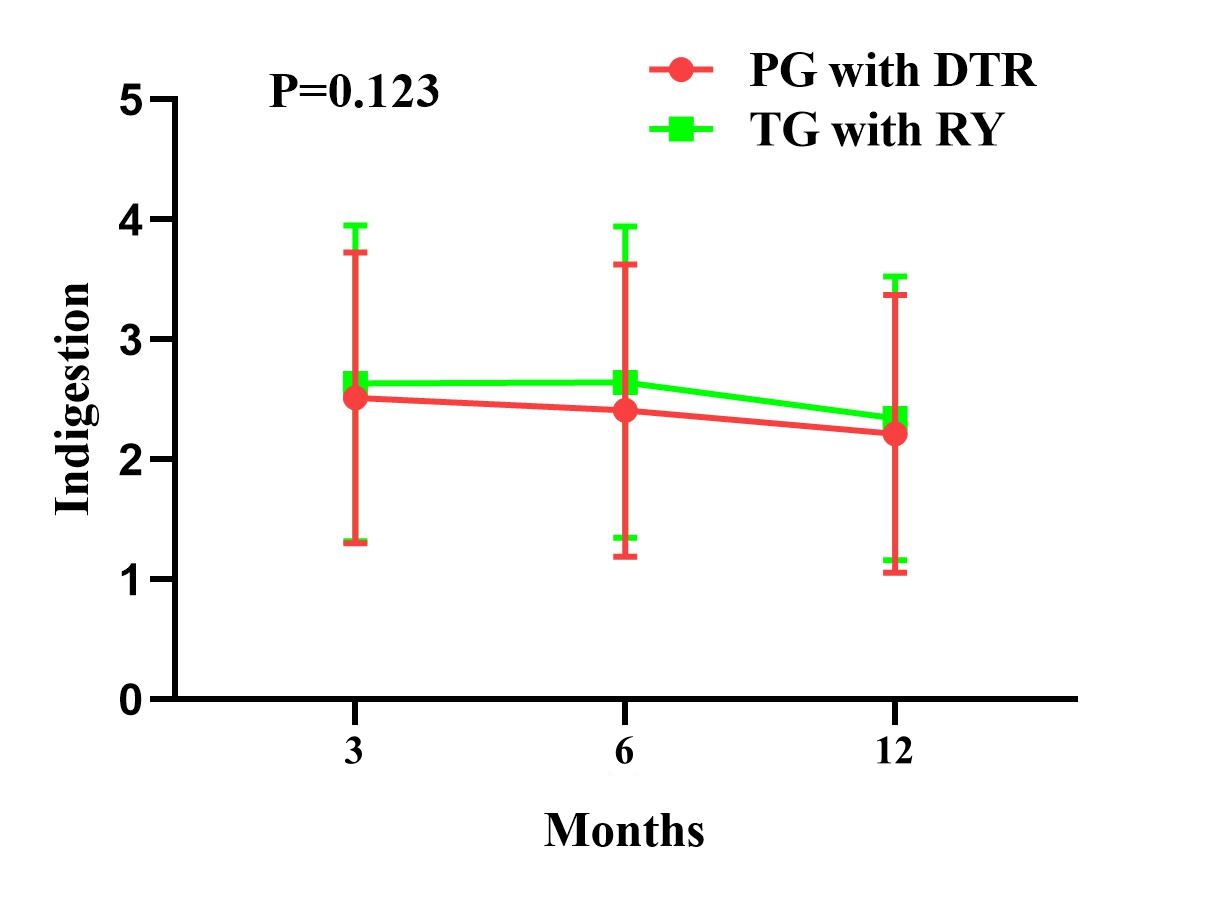
**

**(E)
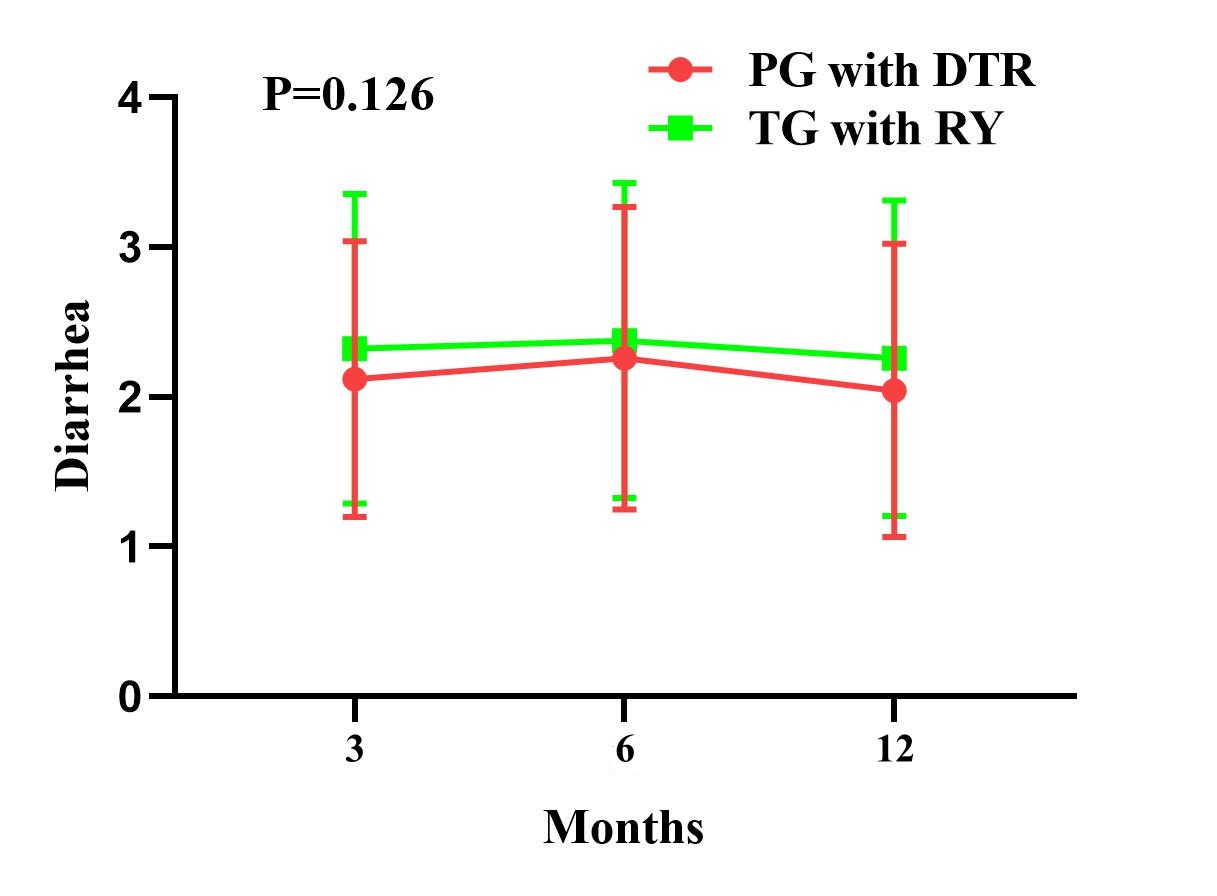
(F)
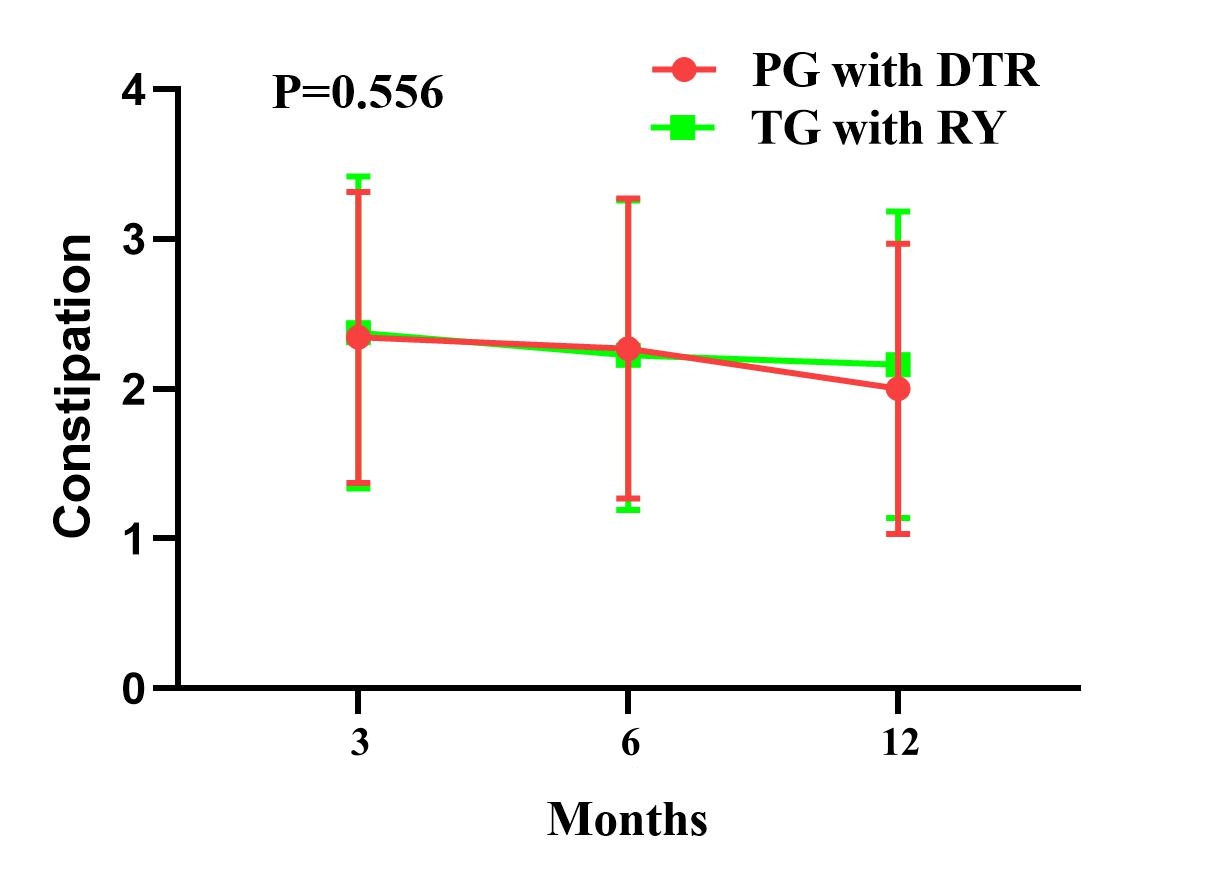
**

**(G)
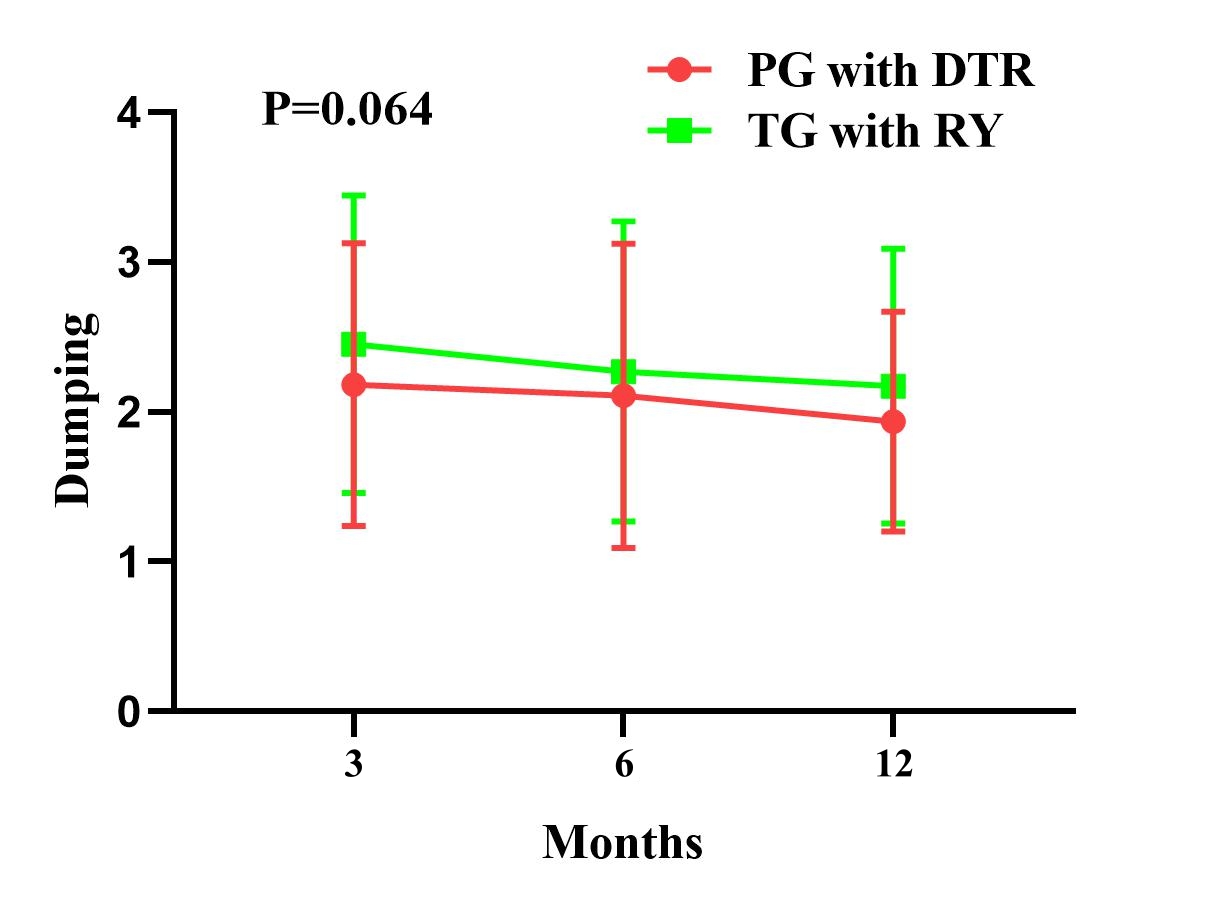
(H)
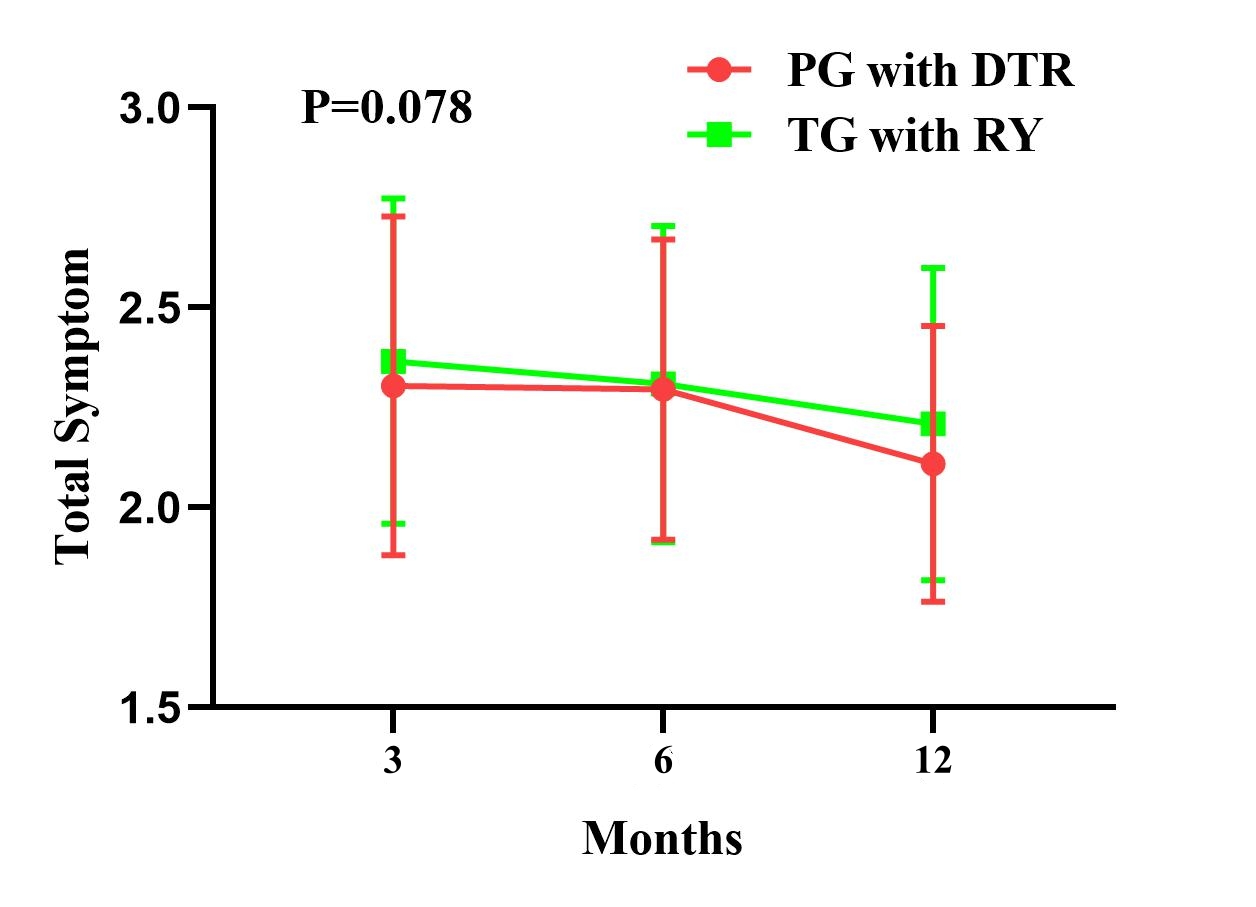
**

**(I)
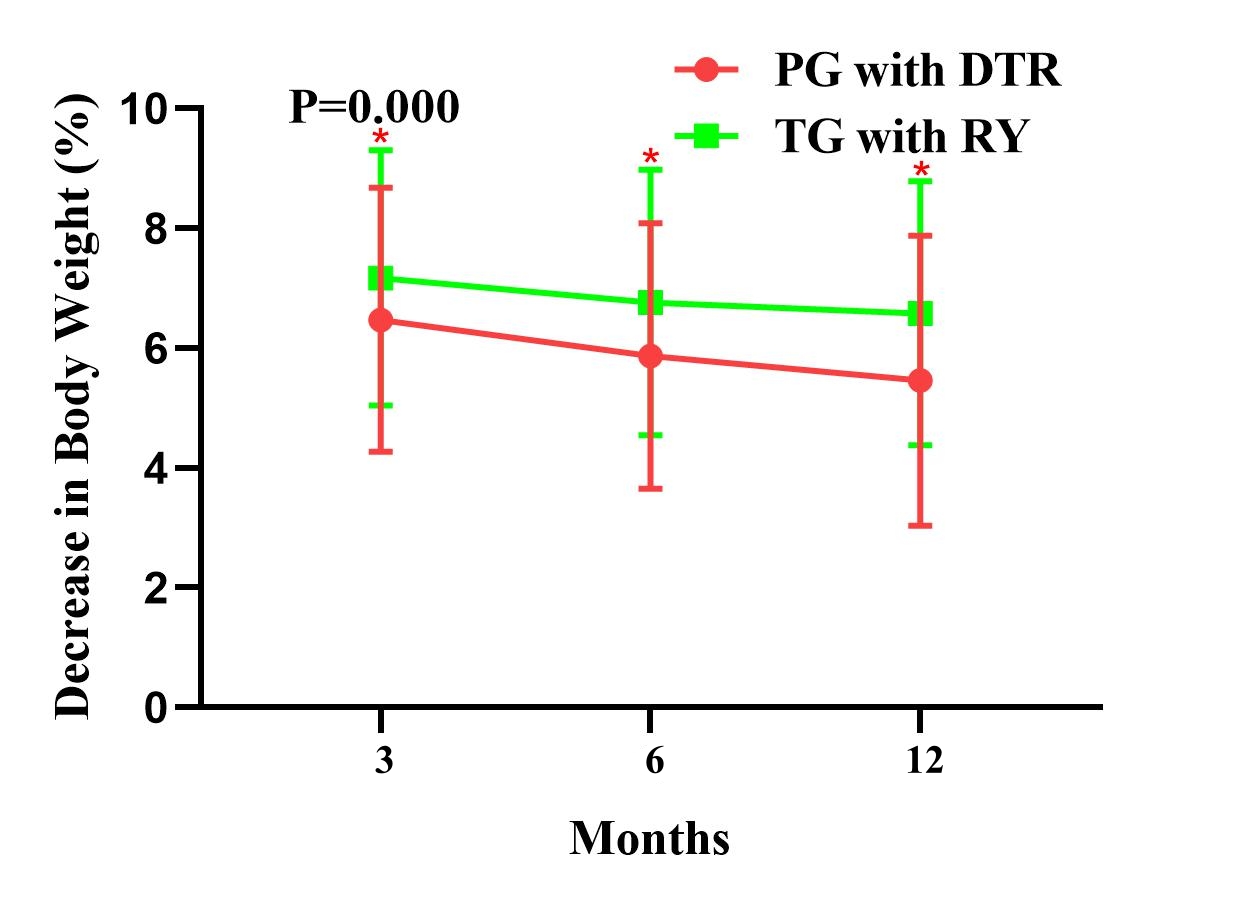
 (J)
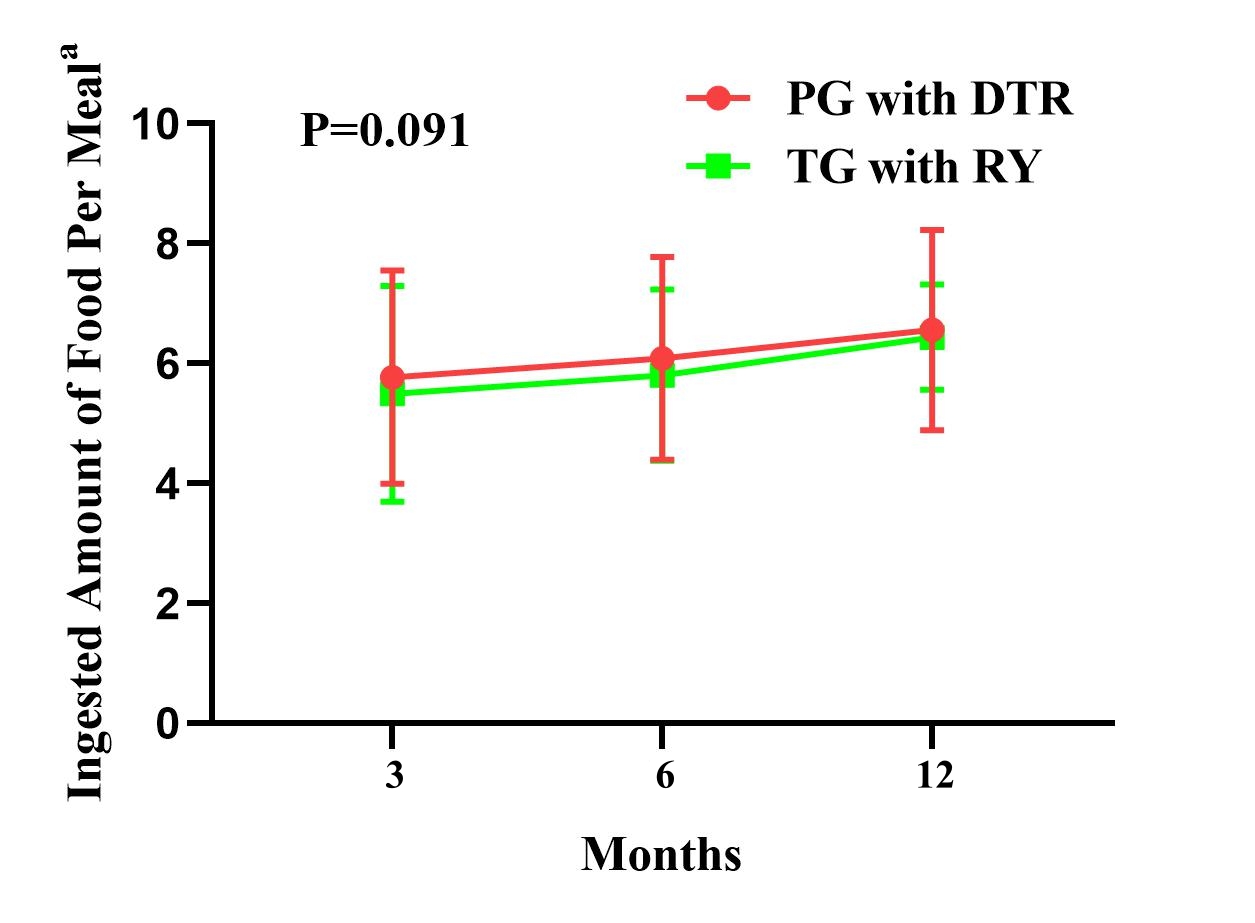
**

**(K)
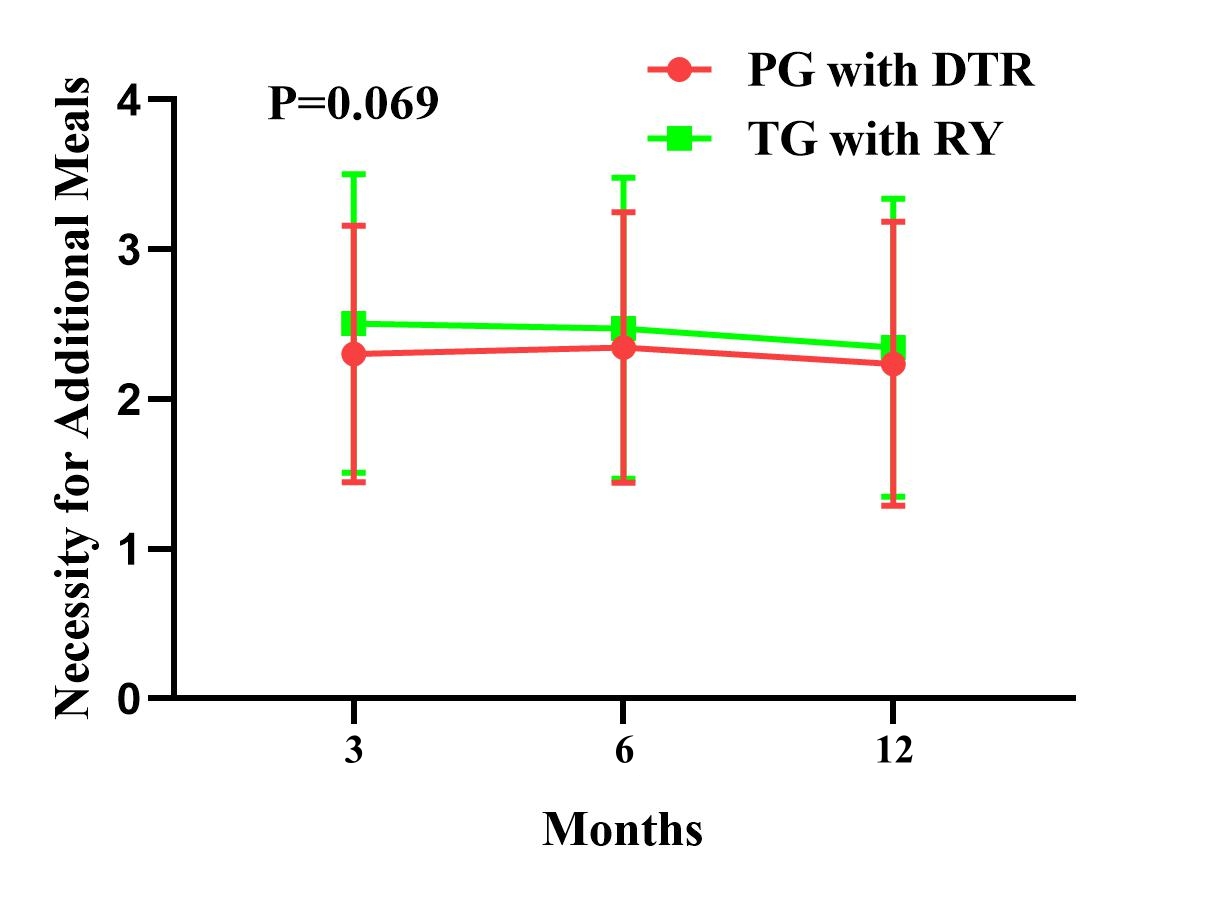
(L)
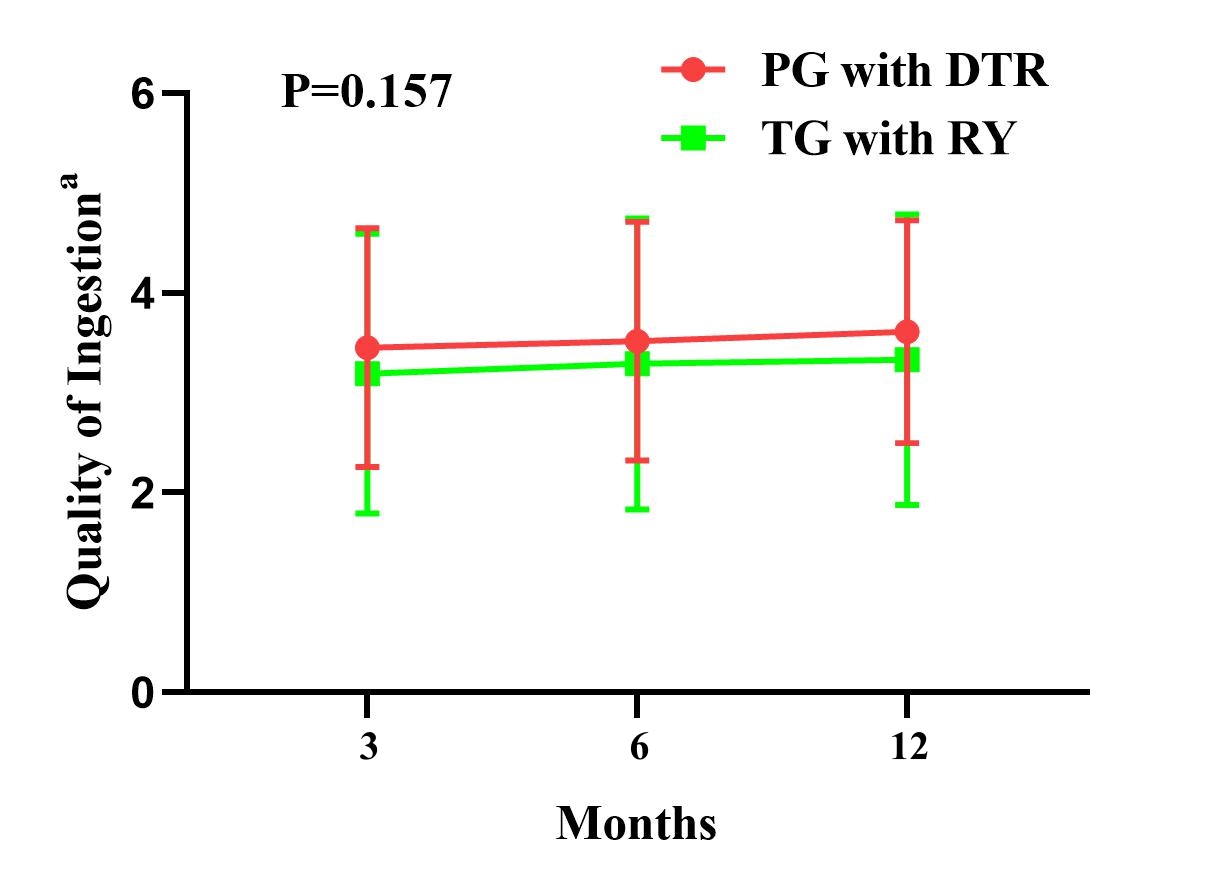
**

**(M)
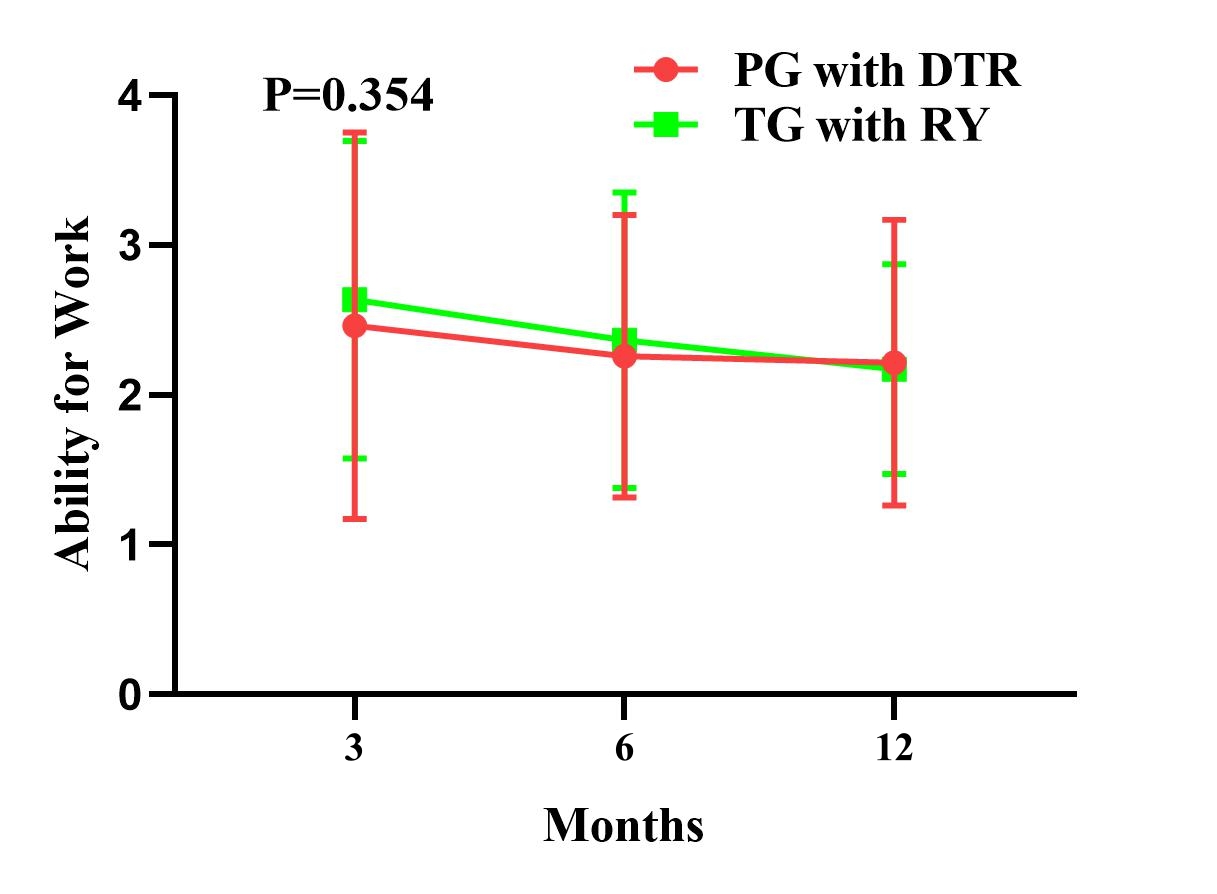
(N)
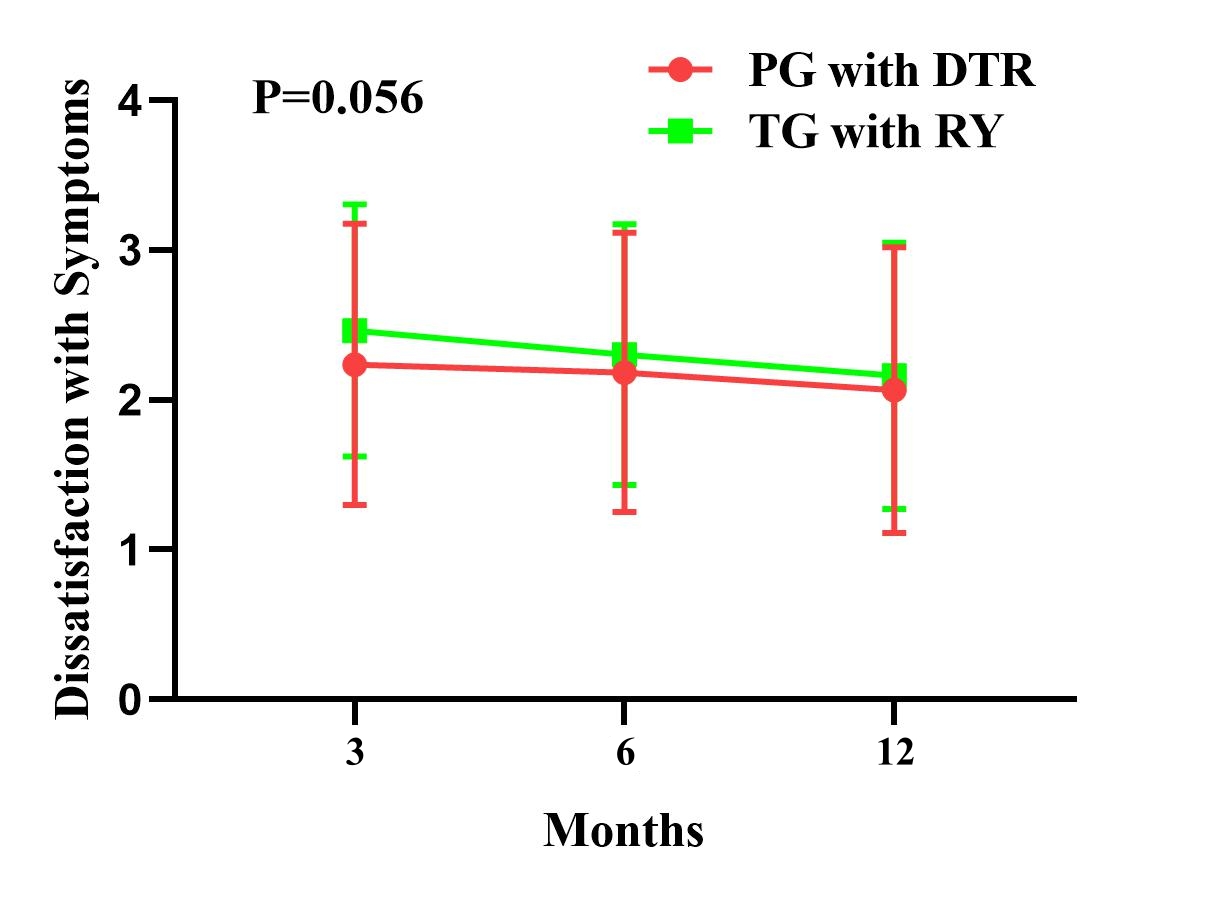
**

**(O)
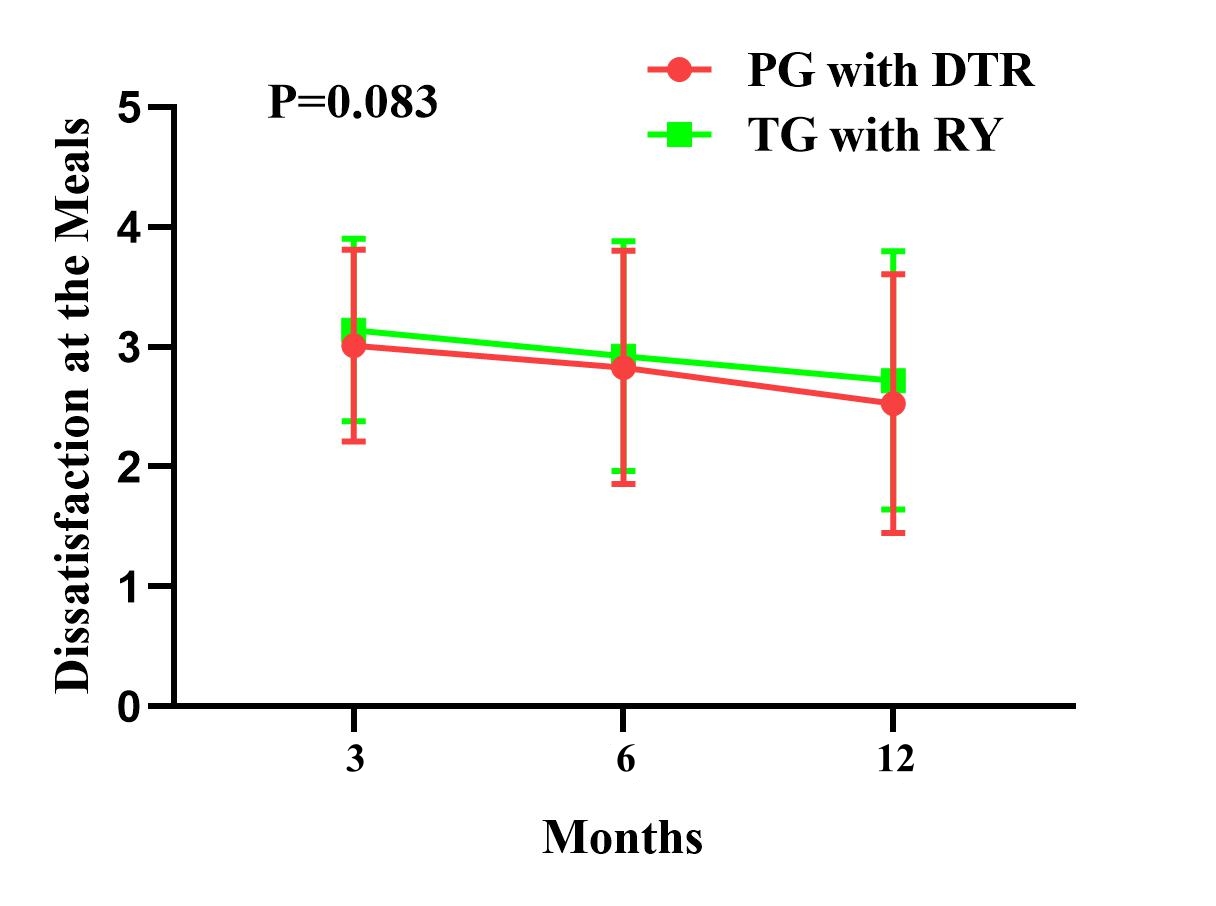
 (P)
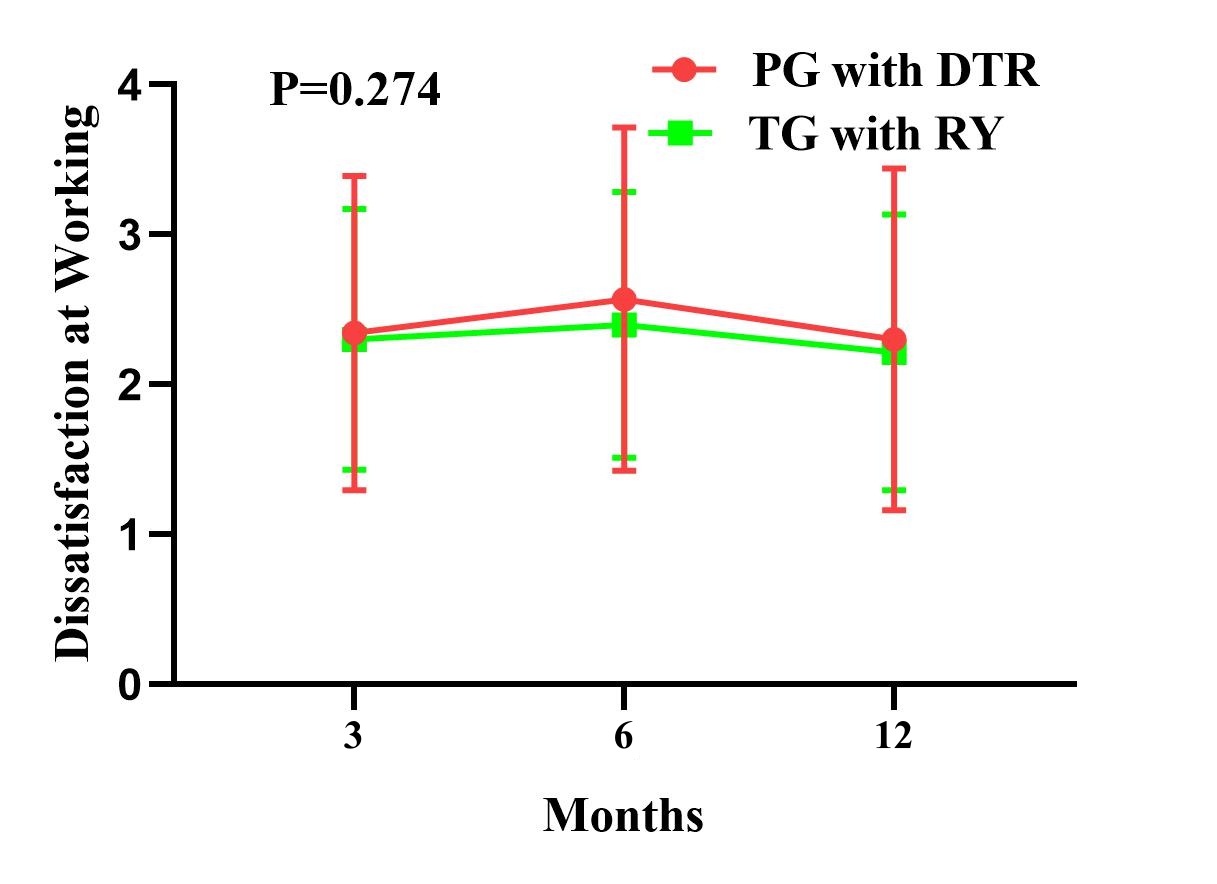
**

**(Q)
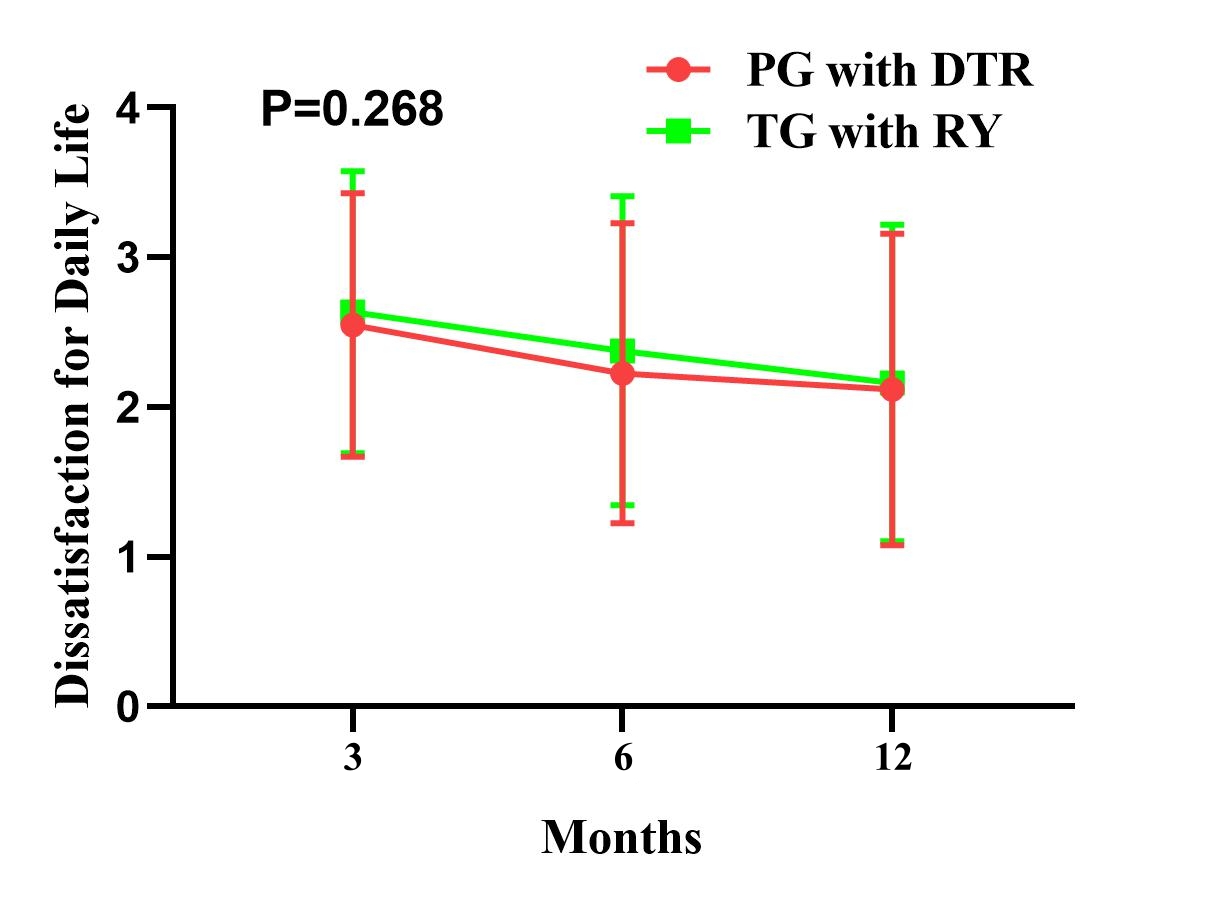
(R)
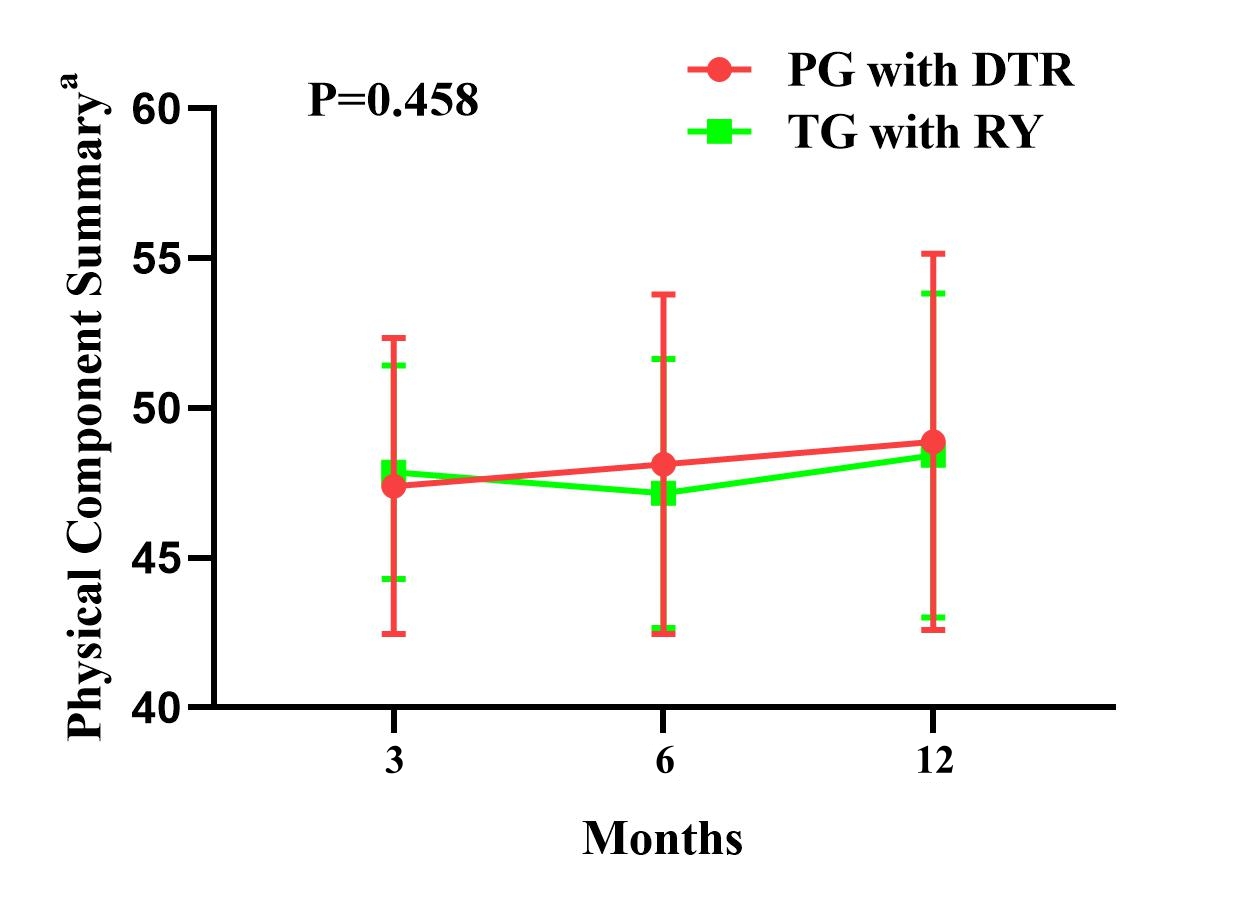
**

**(S)
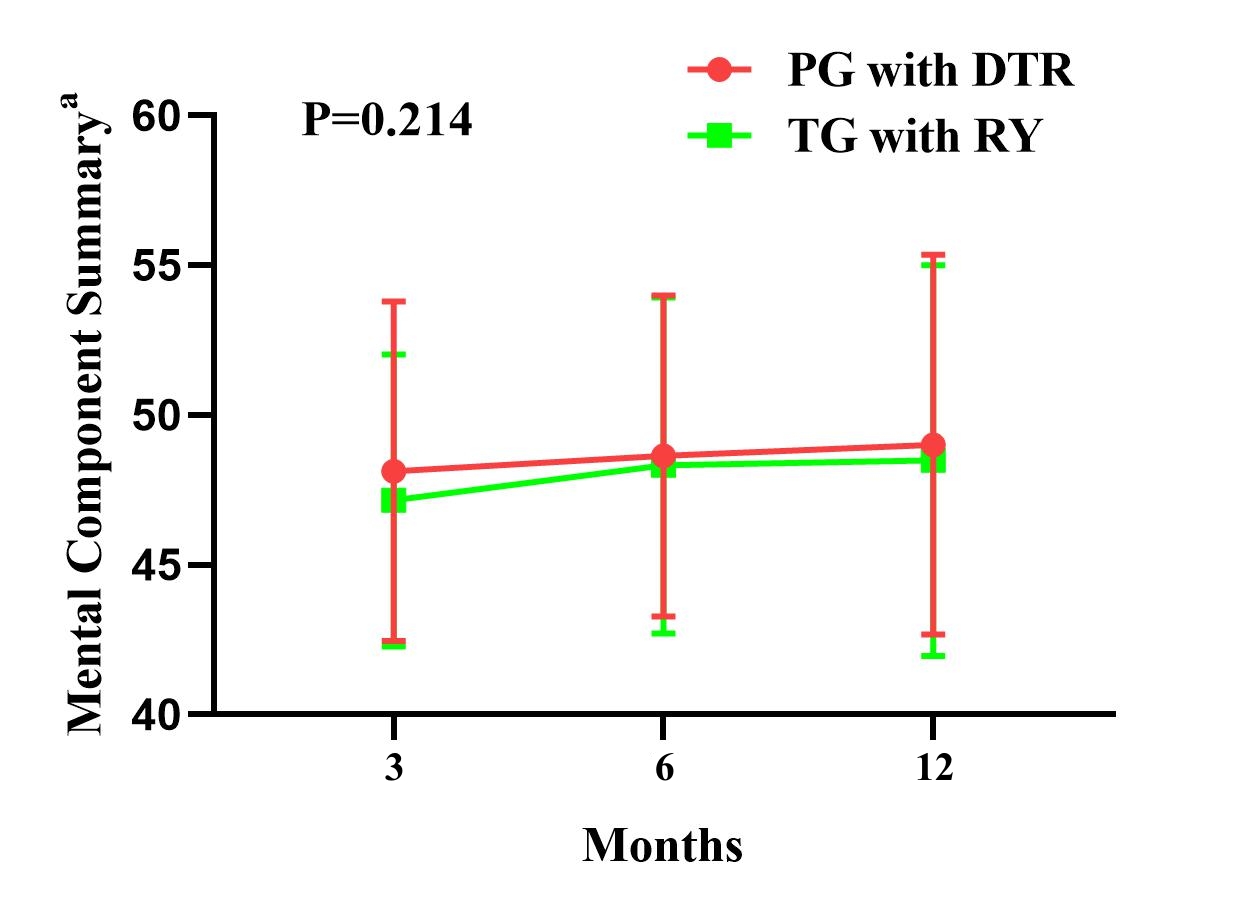
**

SUPPLEMENTARY FIGURES S2 Postoperative QOL assessment by the PGSAS-45. (A) Esophageal reflux; (B) abdominal pain; (C) meal-related distress; (D) indigestion; (E) diarrhea; (F) constipation; (G) dumping; (H) total symptom; (I) decrease in body weight; (J) ingested amount of food per meal; (K) necessity for additional meals; (L) quality of ingestion; (M) ability for work; (N) dissatisfaction with symptoms; (O)dissatisfaction at the meals; (P) dissatisfaction at working; (Q) dissatisfaction for daily life; (R) physical component summary; (S) mental component summary.

^a^higher score indicating better condition.
